# Large strain and high reversible dielectric tunability in Sm-modified PMN-PT ceramics

**DOI:** 10.1038/s41467-026-77914-1

**Published:** 2026-09-18

**Authors:** Hangfeng Zhang, Theo Graves Saunders, Haixue Yan, Yang Hao

**Affiliations:** 1 School of Electronic Engineering and Computer Science, Queen Mary University of London, Mile End Road, London, UK; 2 School of Engineering and Materials Science, Queen Mary University of London, Mile End Road, London, UK

**Keywords:** Actuators, Electronic devices, Chemical engineering, Solid-state chemistry

## Abstract

Relaxor ferroelectrics offer dynamic polar responses desirable for adaptive electronics, yet they are fundamentally constrained by a long-standing trade-off: compositions that deliver large electric-field induced strain typically suffer from pronounced hysteresis, while highly tunable ferroelectrics exhibit limited actuation capability. Here, we demonstrate a crossover composition-tuning strategy that helps balance these competing functionalities within a single perovskite platform. Using qualitative first-principles calculations on selected local-approximant supercells, we examined how Ti substitution affects possible local polarization tendencies in Sm-doped Pb(Mg_1/3_Nb_2/3_)_1-*x*_Ti_*x*_O_3,_ including predominantly 〈110〉-like, mixed multidirectional and 〈001〉-like orientations. Near the relaxor crossover region (*x* = 0.16), the composition exhibits a strain of 0.31% and the highest reversible dielectric tunability of 76.4% under 2 kV mm^−1^ with low remanent polarization and small irreversible tunability. Increasing the Ti content to *x* = 0.22 promotes a more stable polar state and yields a higher strain of 0.54%, but also increases remanent polarization and the irreversible tunability component, reducing the reversible dielectric tunability to 64.0%. Multiscale structural characterization supports composition-dependent evolution toward more spatially coherent local structural and polar responses with increasing Ti content. This work demonstrates composition tuning of local polar correlations near the relaxor crossover regime as an effective strategy for balancing high actuation, dielectric agility, and highly reversible field-induced response within a single compositionally tuned perovskite platform.

## Abstract (web summary)

Relaxor ferroelectrics promise adaptive electronics, but strain and reversible tunability are difficult to combine. Zhang et al. tuned titanium content and local polar correlations in a perovskite to balance strong actuation, dielectric agility, and low hysteresis.

## Introduction

Ferroelectrics with switchable spontaneous polarization underpin a wide range of technologies, including precision actuators^1–3^, ultrasonic transducers^4,5^, high power capacitors^6–9^, and tunable communication devices^10–12^. Yet these diverse functionalities are typically realized in different material systems optimized for specific metrics, such as large piezoelectric coefficients near morphotropic phase boundaries (MPBs)^1,2^, high dielectric permittivity and breakdown strength for capacitors^13,14^, or strong dielectric tunability for frequency-agile devices^10,15,16^. This functional specialization arises from an intrinsic materials trade-off. Compositions near MPB can achieve strong piezoelectric responses through polarization rotation within a flattened free-energy landscape containing multiple competing ferroelectric phases^17–19^, but their long-range ferroelectric domains give rise to pronounced hysteresis and energy dissipation under large signal operation^3,20^. In contrast, ferroelectrics near and just above *T*_c_, which are characterized by nanoscale chemical and polar disorder, exhibit high dielectric tunability and reduced hysteresis^12,21^, yet limited electromechanical performance. Achieving large reversible strain, high dielectric tunability and low hysteresis simultaneously within a single material platform, therefore, remains a central challenge for multifunctional device integration.

Relaxor ferroelectrics are promising candidates to address this limitation because their local chemical heterogeneity, different local polar correlations and facile polarization rotation enable highly responsive polarization^18,19,22,23^. Among them, the solid solution (1-*x*)Pb(Mg_1/3_Nb_2/3_)O_3_-*x*PbTiO_3_ (PMN-PT) has set benchmarks for piezoelectric performance, particularly near the MPB, where the small-signal piezoelectric coefficient (*d*_33_) exceeds that of conventional PZT ceramics^7^. Subsequent compositional engineering and doping have further improved their piezoelectric performance. In particular, incorporating a small amount of rare-earth elements such as Sm^3+^ into the PMN-PT system flattens the free energy landscape and enhances polarization rotation, yielding record-breaking piezoelectric responses^17,24^. Nevertheless, these improvements are mostly focused on PMN-PT (*x* ≥ 0.28) compositions near MPB, where large ferroelectric domains and associated polarization-electric field hysteresis persist. While the *d*_33_ value obtained under low electric fields is commonly used to evaluate quality for piezoelectric sensors, practical actuators typically operate under high fields. Thus, the large-signal effective piezoelectric coefficient, defined as *d*_33_^*^ = *S*_max_/*E*_max_ (where *S*_max_ and *E*_max_ are the maximum strain and electric field, respectively), offers a more relevant and accurate measure of actuator performance. Recent studies have reported high strains exceeding 1% and enhanced *d*_33_^*^ values in MPB systems, but these improvements are accompanied by severe hysteresis losses during field cycling^20,25^.

Conversely, the dielectric tunability is optimized in compositions where dynamic local polar correlations respond reversibly to external bias without domain stabilization. Strategies such as compositional tuning in BaTiO_3_-based ferroelectrics shift *T*_c_ toward ambient conditions to enhance dielectric tunability and reduce dielectric loss, often at the expense of actuation capability^26^. These observations suggest that the key to multifunctionality lies not simply in maximizing polarization magnitude, but in controlling the polarization landscape, such as the distribution, orientation tendency and dynamics of local polar correlations at the crossover between ergodic and nonergodic relaxor ferroelectric states.

In PMN-based relaxor ferroelectrics, dielectric tunability and strain are both sensitive to the underlying polarization state. High dielectric tunability is typically associated with dynamic local polar correlations, whereas large strain involves field-induced polar reorientation, polarization switching, or lattice distortion. Therefore, evaluating both responses together is important for understanding the relaxor ferroelectric crossover and for identifying compositions that best balance actuation capability, dielectric agility, and hysteresis. In this work, we systematically modulate Ti content in Sm-doped PMN-PT (Sm_0.025_Pb_0.9625_(Mg_1/3_Nb_2/3_)_1-*x*_Ti_*x*_O_3_, SPMNT), to identify a composition window that balances reversible dielectric tunability, field-induced strain and low remanent polarization. Qualitative first principles local approximant calculations were used to examine possible changes in local polarization tendencies for selected chemical configurations. The *x* = 0.16 composition lies within the broad room temperature dielectric maximum region and shows reversible dielectric tunability of 76.4% and 0.31% nearly symmetric field-induced strain under 2 kV mm^−1^, while maintaining low remanent polarization and a small irreversible tunability component. These results indicate that composition-dependent modulation of local polar correlations can provide a practical balance between dielectric agility and large-signal actuation within a single perovskite platform.

## Results and discussion

### First-principles insights into local polarization tendencies

**Fig. 1:**
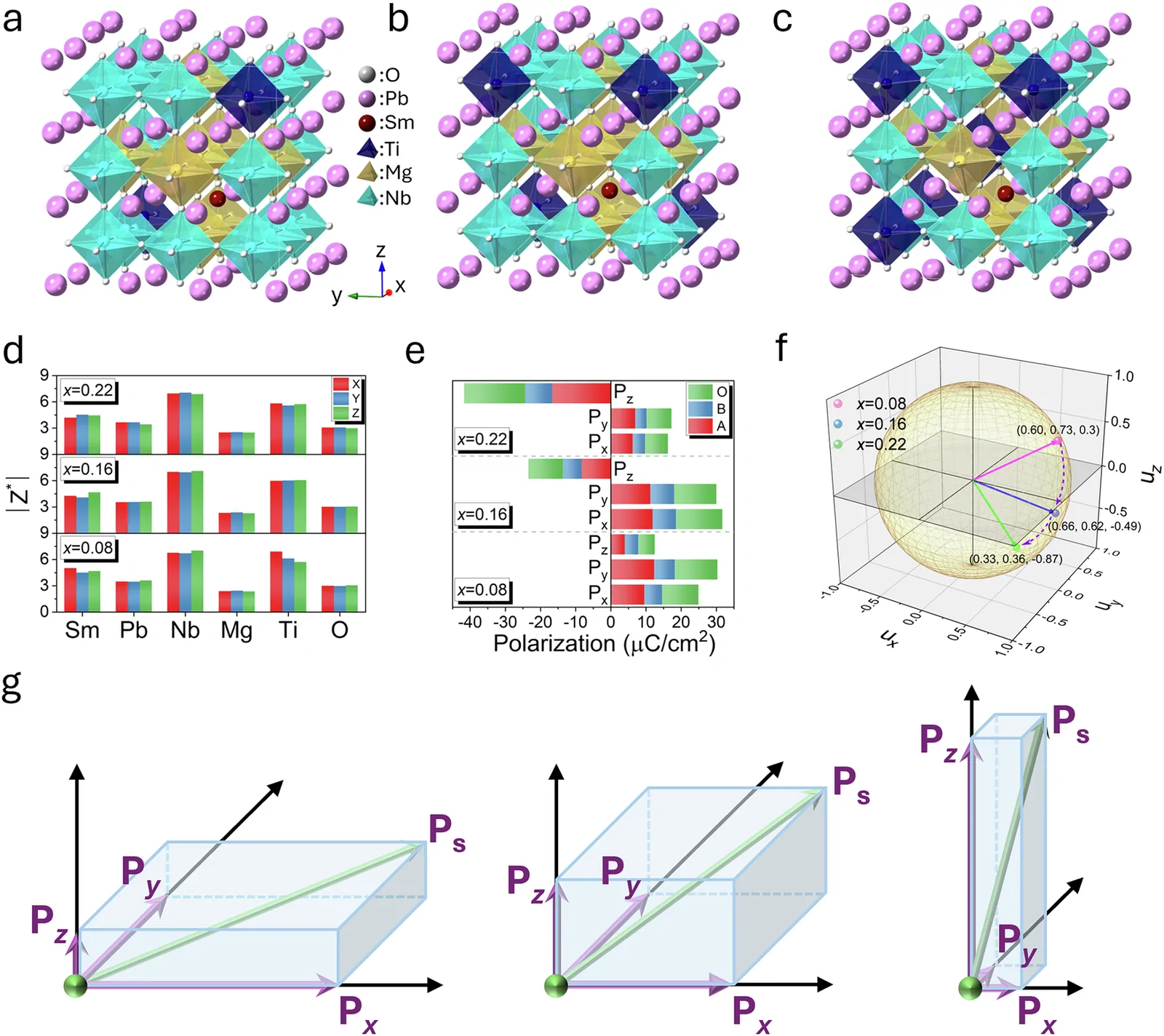
DFT-calculated structures and polarization characteristics of studied compositions in the SPMNT system. DFT-relaxed crystal structures for **a** *x* = 0.08, **b** *x* = 0.16 and **c** *x* = 0.22 compositions. **d** Born effective charge of each element and **e** net polarization along each crystallographic axis for those compositions. **f** 3D visualization of the normalized polarization vectors for *x* = 0.08, 0.16 and 0.22 compositions on a unit sphere. **g** Schematic comparison of the polarization orientations represented by the selected DFT models.

To investigate the local polarization behavior in SPMNT systems, first-principles density functional theory (DFT) calculations were performed on simplified 3 × 3 × 3 local-approximant supercell models representing the nominal *x* = 0.08, 0.16 and 0.22 compositions (Fig. 1a–c). The XRD-derived lattice parameters were used as constraints, so that the calculations could examine possible local polarization tendencies within the experimental structural framework. The calculated average Born effective charges (Z^*^) show only minor directional variation in the selected models (Fig. 1d). The A-site cations exhibit enhanced Born effective charges (|Z^*^| > 4 for Sm and |Z^*^| > 3 for Pb) compared to their nominal valence states (Sm^3+^ and Pb^2+^), indicating that A-site off-center displacements contribute significantly to the polarization. Among the B-site cations, Nb and Ti ions also show increased Born effective charges, consistent with B–O hybridization involving *d* orbitals with O 2*p* states, whereas Mg shows a slight increase in |Z^*^|, suggesting a more ionic bonding character. The oxygen anions show enhanced |Z^*^| value, compensating for the charges of cations within the lattice. (Fig. 1e). For the representative DFT models with different Ti contents, the calculated polarization arises from coupled displacements of the A-site, B-site and oxygen sublattices. Although the Pb/Sm and oxygen sublattices make substantial contributions, B-site off-centering and BO_6_ octahedral distortion play an important role in tuning the polarization tendency.

The calculated polarization vectors and unit vectors provide a local-approximant illustration of differences in local polarization tendencies among the selected configurations at different Ti contents (Fig. 1f). In the *x* = 0.08 model, the spontaneous polarization vector (P_s_) lies mainly in the x-y plane, approximately along a pseudocubic 〈110〉-like direction (Fig. 1g). In the *x* = 0.22 model, the polarization vector tilts more strongly toward the z-axis, showing a tendency closer to a 〈001〉-like direction. The *x* = 0.16 model exhibits more comparable polarization components along all three crystallographic directions, suggesting a more balanced local polarization tendency. To further assess this behavior, seven additional *x* = 0.16 configurations were relaxed, and their polarization directions were calculated. The resulting vectors span a broad orientational range between those of the representative *x* = 0.08 and *x* = 0.22 models (Supplementary Fig. 1), indicating that the calculated polarization direction is sensitive to the local Mg/Nb/Ti arrangement. To clarify the effect of Sm doping, an additional DFT calculation was performed for undoped PMNT08, which remains cubic experimentally (Supplementary Fig. 1). The calculated polarization of PMNT08 is dominated by a large P_z_ component, indicating a strong 〈001〉-like polarization tendency in this representative model. After Sm substitution, the P_z_ dominance is reduced, and the polarization components are redistributed, indicating that Sm substitution can modify the local polarization tendency.

### Crystal structure and dielectric behavior of relaxor ferroelectrics

**Fig. 2:**
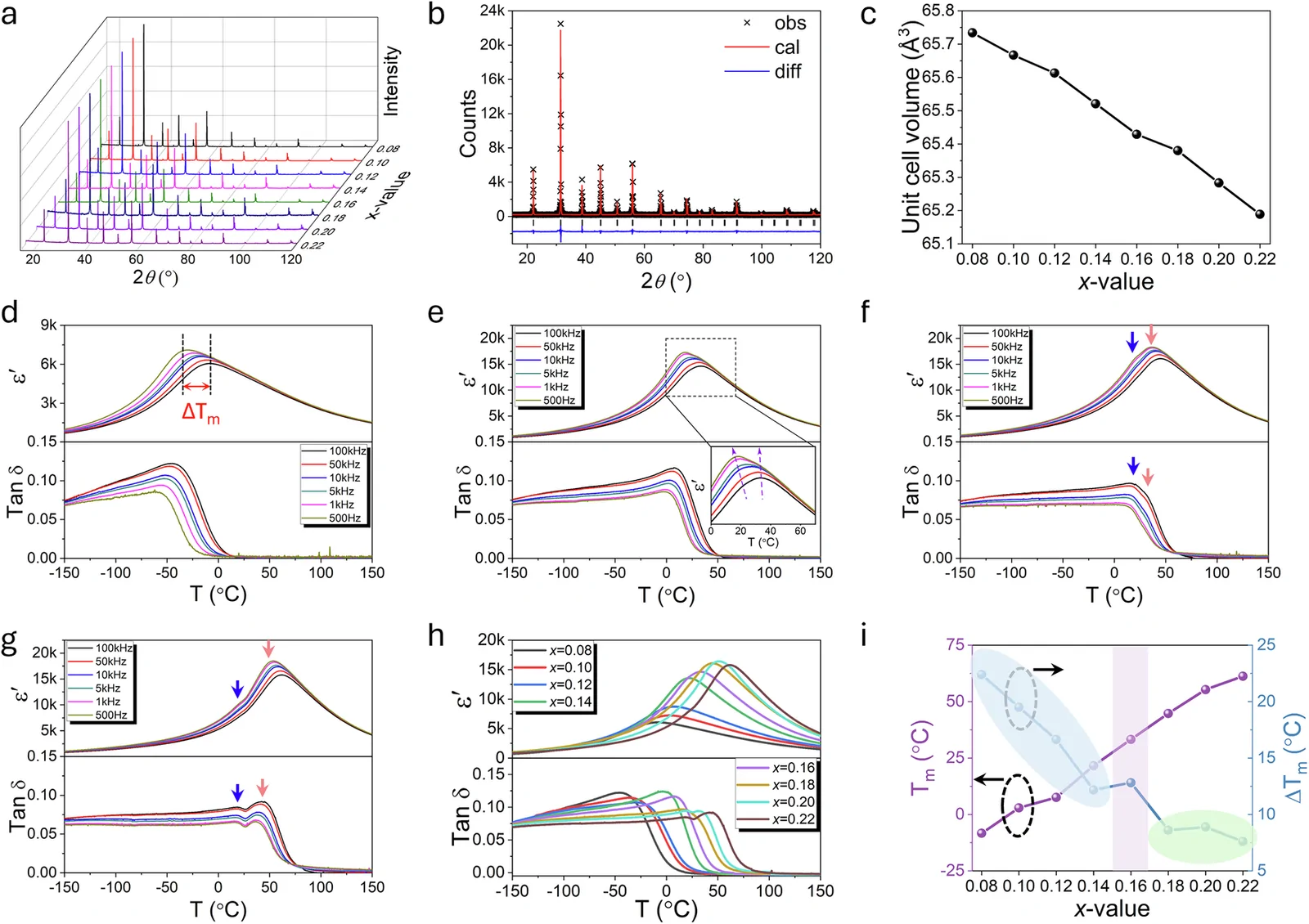
Structure and dielectric properties of studied compositions in the SPMNT system. **a** Normalized XRD pattern measured at room temperature for the studied compositions. **b** Fitted XRD profiles for *x* = 0.16 composition. **c** Compositional dependence of unit cell volume for SPMNT system. Temperature dependence of dielectric permittivity (ε') and loss tangent (tan δ) for **d** *x* = 0.08, **e** *x* = 0.16 and **f** *x* = 0.18 and **g** *x* = 0.22 compositions. **h** Comparison of dielectric responses at 100 kHz for the studied system and **i** compositional dependence of maximum permittivity temperature (*T*_m_) and the corresponding temperature dispersion (∆*T*_m_).

XRD patterns of the SPMNT (*x* = 0.08–0.22) ceramic powder, showing nearly identical diffraction patterns for studied compositions, confirm the formation of a single perovskite structure without detectable secondary phases (Fig. 2a). Rietveld refinements using a cubic perovskite model with space group *Pm*−3*m* reveal good agreement between observed and calculated profiles (Fig. 2b and Supplementary Fig. 2), indicating the average structure of non-polar symmetry. The details of refined parameters for studied compositions are summarized in Supplementary Table 1. The refined unit cell volume exhibits an almost linear decrease with increasing Ti concentration (Fig. 2c). The shrinkage in unit cell volume is attributed to the smaller ionic radius of Ti^4+^ (0.605 Å, coordination number, CN = 6) compared to the average radius of (Mg_1/3_Nb_2/3_)^4+^ (0.666 Å, CN = 6)^27^. To further study the effect of Sm doping and A-site vacancy, undoped Pb(Mg_1/3_Nb_2/3_)_1-*x*_Ti_*x*_O_3_ compositions with *x* = 0.08, 0.16, and 0.22 and the A-site deficient Pb_0.9875_(Mg_1/3_Nb_2/3_)_0.84_Ti_0.16_O_3_, denoted as PMNT08, PMNT16, PMNT22 and PMNT16v, were also prepared and their refined XRD profiles are shown in Supplementary Fig. 2. PMNT08 was refined using the cubic *Pm*−3*m* perovskite model, whereas PMNT16, PMNT22 and PMNT16v were fitted using the orthorhombic *Amm*2 ferroelectric model, consistent with their dielectric and *P–E* behavior. This comparison indicates that, in the undoped PMNT system, increasing Ti content promotes the development of a stable ferroelectric phase, while the SPMNT compositions preserve an average cubic structure over the same compositional range. The result suggests that Sm doping helps destabilize long-range ferroelectric ordering and maintains a more disordered relaxor-like average structure. SEM images show that the studied compositions possess dense microstructures with relative densities above 95% and average grain sizes of approximately 3.9–9.1 μm (Supplementary Fig. 3). Dielectric spectra of all compositions exhibit a broad dielectric permittivity maximum *T*_m_ and a corresponding loss tangent peak (Fig. 2d–g and Supplementary Fig. 4). The dielectric permittivity peak shows significant frequency dispersion for compositions with low *x*-value, and their *T*_m_ shifting to higher temperatures with increasing measurement frequency. Such behavior corresponds to an ergodic relaxor state, where dynamic local polar correlations dominate the dielectric response and exhibit a wide distribution of relaxation times^28,29^. For the undoped PMNT system, PMNT08 shows a broad permittivity maximum with a *T*_m_ of *ca*. 20 °C and clear frequency dispersion, indicating relaxor-like behavior. In contrast, PMNT16 and PMNT22 exhibit dielectric maxima at *ca*. 98 °C and 126 °C, respectively, with weaker frequency dispersion, consistent with the development of a more stable ferroelectric phase. This is also in agreement with their room temperature *Amm*2 structural refinements and with the reported orthorhombic/monoclinic-like ferroelectric distortions in PMN-PT-based compositions^30,31^. By comparison, PMNT16v composition shows a lower *T*_m_ of 87 °C with stronger frequency dispersion of the dielectric peaks, suggesting that A-site vacancies disrupt long-range ferroelectric ordering and promote a more relaxor-like response. The frequency dependence of the permittivity peak gradually decreases with increasing Ti concentration up to *x* = 0.14, as evidenced by the decreasing ∆*T*_m_ (temperature difference in *T*_m_ at 500 Hz and 100 kHz, Fig. 2i). For higher Ti concentration (*x* > 0.16), the dielectric peak remains relatively broad, but the frequency dispersion is much lower and constant with increasing Ti concentration. Notably, for compositions with *x* ≥ 0.16 (Fig. 2e–g and Supplementary Fig. 4), an additional dielectric anomaly appears on the low-temperature side of the main permittivity peak, accompanied by a shoulder feature in the dielectric loss. Such anomalies have been seen in PMN-PT-based relaxors and are commonly associated with the emergence of competing local polar distortions with symmetries suggestive of orthorhombic and tetragonal states, as frequently observed in PMN-based relaxors^17,32,33^. The emergence of this feature indicates the onset of enhanced local polar correlations, where the energy barriers between different polar orientations begin to increase. This suggests a gradual transition from an ergodic state toward localized symmetry-breaking behavior. Although the average structure remains cubic, these features indicate enhanced local polar correlations and reduced polar reversibility at higher Ti contents^31,34^. With increasing Ti concentration, the collinear state, corresponding to the temperature region between the anomaly and the main permittivity peak, extends over a broader temperature range, supported by temperature dependence of dielectric permittivity in Fig. 2h. This behavior may be associated with a reduced free energy barrier between competing orthorhombic and tetragonal local polar states, which facilitates field-induced reorientation within local polar correlations^17,32^. These findings are qualitatively consistent with the selected DFT models, in which the representative higher Ti configuration shows a stronger 〈001〉-like polarization tendency.

### Field-induced functional response and switching dynamics

**Fig. 3:**
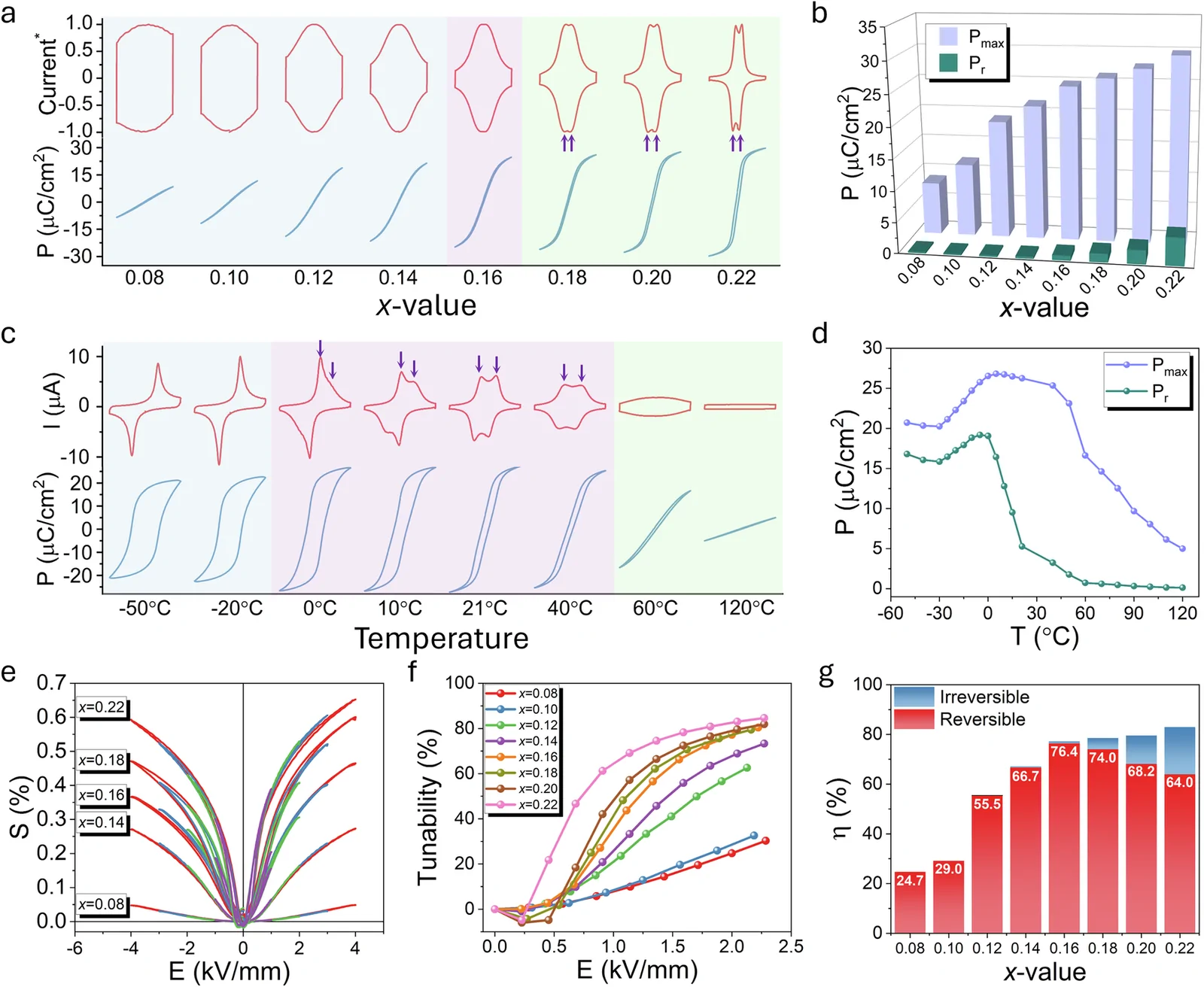
Ferroelectric switching behavior and functional responses of the studied SPMNT compositions. **a** Normalized current (*I*/*I*_max_)-electric field and *P-E* loops measured under 2 kV mm^−1^ at 1 Hz for studied SPMNT compositions. **b** Compositional dependence of maximum polarization (P_max_) and remanent polarization (P_r_). **c** Temperature dependence of *I-E*/*P-E* loops measured under 1 kV mm^−1^ for *x* = 0.22 composition. **d** Temperature dependence of P_max_ and P_r_ for *x* = 0.22 composition. **e** Bipolar *S–E* loops for selected SPMNT compositions under different electric fields. **f** Tunability variation under different DC bias for the SPMNT compositions. **g** Reversible and irreversible tunability at 100 kHz under DC bias of 2 kV mm^−1^ for the SPMNT systems.

Ferroelectricity measurements were performed to examine the evolution of the polarization switching behavior and functional properties in SPMNT ceramics (Fig. 3). For low Ti compositions (*x* ≤ 0.12), the polarization-electric field (*P–E*) loops are narrow, and the current peaks in current-electric field (*I–E*) loops are weak (Fig. 3a), indicating polarization response dominated by highly dynamic and localized polar correlations, characteristic of an ergodic relaxor state. As the Ti content increases to *x* = 0.16, the nonlinear *P–E* hysteresis loops become more pronounced with higher polarization response, suggesting enhanced magnitude of polarization and increased configurational diversity. For higher Ti concentrations (*x* ≥ 0.18), multiple switching peaks appear in the *I–E* loops, indicating a more complex multi-step polarization switching process associated with the stronger field-induced ordering of local polar correlations^35,36^. While maximum polarization (P_max_) increases with increasing Ti content, the remanent polarization (P_r_) remains negligible for *x* ≤ 0.16 and begins to increase at higher Ti concentration (Fig. 3b), indicating increased stability of field-induced polar order after removal of the field. For compositions with *x* ≥ 0.16, further increases in polarization above *ca*. 2 kV mm^−1^ are small (Supplementary Fig. 5), suggesting the formation of field-aligned and highly responsive dipolar configurations. For the undoped PMNT system, PMNT08 exhibits relatively high maximum polarization in the *P–E* loop with current peaks near zero field in the *I–E* loop. In addition, compared with SPMNT16 and SPMNT22, PMNT16, PMNT22 and PMNT16v show much sharper switching current peaks in the *I–E* loops and typical ferroelectric *P–E* loops with clear remanent polarization, indicating a more stable ferroelectric response. The temperature dependence of *I–E*/*P–E* loops of *x* = 0.22 composition was studied because this composition exhibits clear switching-current features, making it suitable for tracking the evolution of polarization switching with temperature. These measurements reveal a progressive transition from a ferroelectric state at low temperature to relaxor-ferroelectric behavior at higher temperature (Fig. 3c and Supplementary Fig. 6). At temperatures below 0 °C, the two sharp current peaks together with the typical ferroelectric *P–E* hysteresis loops indicate conventional domain switching and stable long-range ferroelectric order. As the temperature increases to 0–10 °C, two additional current peaks occur beside the main current peaks and the hysteresis loop becomes narrower, suggesting a more complex multi-step switching process associated with partial field-induced ordering of local polar correlations, characteristic of a nonergodic relaxor state^37^. When the temperature increases above 21 °C, current peaks appear in each quadrant of *I–E* loops and gradually shift toward higher electric fields, which is attributed to a reversible field-induced transition between a weakly ordered relaxor state and a field-aligned polar state. During field loading, dynamic or weakly correlated local polar correlations are driven into a more field-aligned polar state, while during field unloading, the induced polar state relaxes back toward the initial relaxor state, producing additional current peaks. The transition from nonergodic to ergodic state is also consistent with the anomaly (*~*20 °C) observed in the dielectric spectrum. This transition is further supported by the temperature variation of P_max_ and P_r_ (Fig. 3d), where P_r_ decreases rapidly between 0 and 21 °C while P_max_ remains relatively high. At higher temperatures, both P_max_ and P_r_ decrease significantly and current peaks merge into broad features at 60 °C, consistent with the *T*_m_ (*~*61 °C). Further increasing the temperature to 120 °C, the *P-E* loop becomes nearly linear and switching current peaks disappear, as the material approaches a complete paraelectric state. Meanwhile, the strain-electric field (*S–E*) loops reveal nearly symmetric, sprout-shaped curves for *x* = 0.08, 0.16 and 0.22 compositions, indicative of reversible strain behavior (Fig. 3e). The maximum strain value (*S*_max_) increases with Ti concentration, reaching 0.67% and 0.46% at 4 kV mm^−1^, for *x* = 0.22 and 0.16, respectively. At a driving field of 2 kV mm^−1^, *S*_max_ values of 0.31% and 0.54% are observed for *x* = 0.16 and 0.22 compositions, corresponding to *d*_33_^*^ of approximately 1550 and 2700 pm V^−1^, respectively. Slight asymmetry in S_max_ under positive and negative fields could be associated with a weak electromechanical bias arising from asymmetric field-induced polar reorientation, residual stress, or defect structures such as oxygen-vacancy-related defect dipoles^20^. This high electromechanical response in *x* = 0.22 composition could be attributed to the stronger field-induced polar reorientation and increased stability of local polar coherence. The *P–E* and *S–E* loops remain nearly unchanged from 0.5 Hz to 10 Hz, indicating that the polarization switching and electromechanical strain are dominated by fast reorientation of local polar correlations (Supplementary Fig. 7). Furthermore, the *x* = 0.16 composition maintains a large strain of 0.29% after 100 cycles. The observed decrease is likely attributed to localized pinning of polar correlations by internal bias fields or defect dipoles.

In general, the increase in dielectric tunability with applied bias electric field is attributed to the suppression of dipole vibrations and the field-induced reorientation of local polarization (Fig. 3f). For compositions with *x* > 0.16, an anomalous decrease in tunability is observed at low electric fields, which is associated with a field-induced phase transition that leads to a temporary increase in dielectric permittivity^17,32^. This is consistent with the observation of four current peaks in *I–E* loops. At 2 kV mm^−1^, commonly used in commercial applications^20^, total dielectric tunability increased rapidly with Ti content up to *x* = 0.16 and remains high for *x* ≥ 0.16 (Fig. 3g). The total tunability of the *x* = 0.16 composition at 2 kV mm^−1^, measured using six independently prepared specimens, was 78.2 ± 1.0%. The reversible component reaches its maximum at *x* = 0.16 composition. With further Ti substitution, the irreversible tunability component increases, leading to a reduction in reversible tunability despite the high total tunability. The reversible tunability values of 76.4% for *x* = 0.16 and 64.0% for *x* = 0.22 are representative values obtained from the reversible/irreversible tunability separation shown in Fig. 3g. This trend is consistent with the evolution of remanent polarization and small-signal piezoelectric coefficient *d*_33_ shown, where both remain low up to *x* = 0.16 but increase significantly at higher Ti contents. The studied SPMNT compositions show current densities within the range of 20–50 μA cm^−2^ (Supplementary Fig. 8). The best-balanced performance of *x* = 0.16 is attributed to the combined effect of room temperature falling within the broad dielectric maximum region around *T*_m_, together with its location on the reversible side of the Ti-induced polar state crossover. In this composition, local polar correlations are sufficiently developed to give strong dielectric and strain responses but remain highly reversible without strong remanent polar stabilization. With further Ti substitution, as in *x* = 0.22, the polar state becomes more stabilized, producing larger strain but also higher remanent polarization and a larger irreversible tunability component. This behavior is consistent with field-induced relaxor-to-ferroelectric or polar-ordering transitions reported in PMN-PT-based relaxor systems^38–40^.

### Multiscale characterization of local polar correlations with Ti substitution

**Fig. 4:**
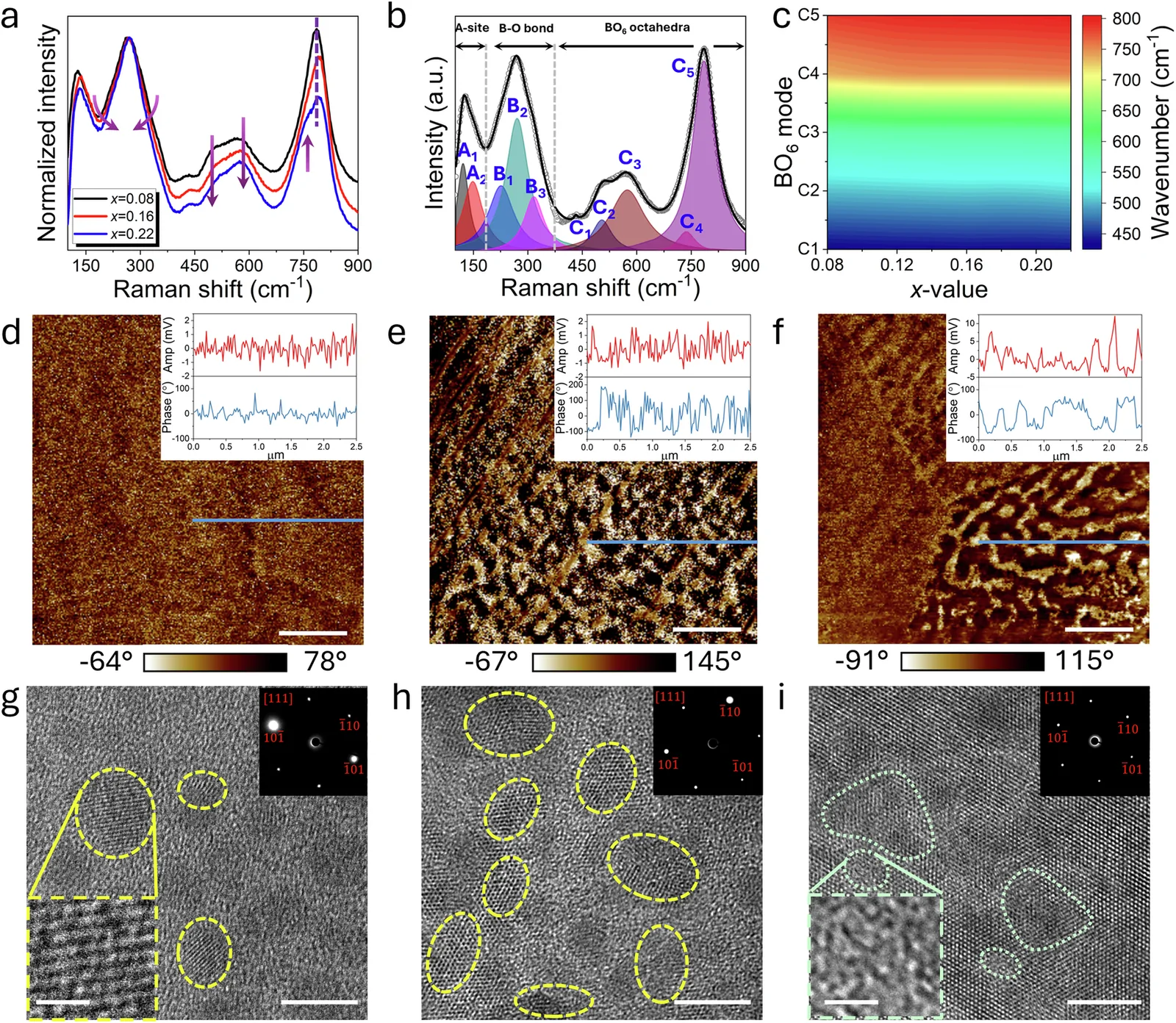
Local structural and polar correlation characterization of selected SPMNT compositions. **a** Raman spectra of normalized intensity for *x* = 0.08, 0.16 and 0.22 compositions. **b** Fitted Raman spectrum for *x* = 0.08. **c** Compositional dependence of the Raman peaks of the BO_6_ mode. PFM phase images for **d** *x* = 0.08, **e** *x* = 0.16 and **f** *x* = 0.22 compositions (insets show the corresponding phase and amplitude line profiles). HRTEM images along the [111] zone axis for **g** *x* = 0.08, **h** *x* = 0.16 and **i** *x* = 0.22 compositions. The dashed yellow circle and light green circle regions represent relatively coherent and less coherent lattice contrast, respectively. Scale bars: 1 µm in (**d**–**f**), 5 nm in (**g**–**i**), and 1 nm in the magnified HRTEM insets in (**g**, **i**).

Raman spectra of *x* = 0.08, 0.16 and 0.22 compositions reveal clear changes with increasing Ti concentration, including peak shifts and broadening, indicating the evolving local structural distortions (Fig. 4a). In particular, the shoulder peak at 740 cm^−1^, which is commonly associated with enhanced polar order in relaxors, becomes more pronounced with higher Ti concentration. The Raman modes were deconvoluted into ten Lorentzian components (Fig. 4b and Supplementary Fig. 9) and grouped into three characteristic vibrational regions: Region A ( ≤ 200 cm^−1^) linked to A-site cation motion, Region B (200–400 cm^−1^) corresponding to B–O bond vibrations, and Region C (>400 cm^−1^) representing BO_6_ octahedral modes. Notably, the dominant C modes harden (shift to higher wavenumber) with increasing Ti content (Fig. 4c), indicating stiffened B–O bonding and more rigid BO_6_ octahedra, consistent with reduced dynamic distortion^34,41–43^. Accordingly, the evolution of the Raman spectra indicates a gradual increase in local polar coherence with increasing Ti content. The hardening of the C-modes and a strengthened 740 cm^−1^ peak suggest increased local polar coherence and a reduced degree of dynamic octahedral distortion, consistent with the ferroelectric and dielectric results.

Topography images of PFM show smooth and well-polished surfaces with root mean square roughness (R_q_) of 4.43, 2.80 and 5.05 nm for *x* = 0.08, 0.16 and 0.22 compositions (Supplementary Fig. 10), respectively. For *x* = 0.08, the phase image shows a diffuse contrast with small phase and amplitude fluctuations, indicating weak local polar piezoresponse. For the *x* = 0.16 composition, the PFM phase image shows a stronger and more spatially heterogeneous phase contrast, indicating enhanced local polar response. For *x* = 0.22 composition, the phase contrast becomes more continuous and better defined, indicating increased spatial coherence and stability of the local polar response. The relatively large and stable phase fluctuation along the line profile is consistent with stronger local polar coherence. The three distinct phase-contrast regions observed for *x* = 0.22 are attributed to the three grains within the scanned region, each exhibiting a different piezoelectric response due to the crystallographic orientation differences. The PFM results indicate that increasing Ti content enhances the spatial coherence and stability of the local polar response, while *x* = 0.16 retains a more heterogeneous and reversible local polar state compared with *x* = 0.22^31,34^. High-resolution transmission electron microscopy (HRTEM) along the [111] crystallographic zone axis provides direct atomic-scale insight into the evolution of local polar structure. In *x* = 0.08 composition, only limited local lattice-contrast variations are observed, consistent with a weak local polarization response and a broad current peak observed in the *I–E* loop. With increasing Ti content at *x* = 0.16, local lattice-contrast variations become more pronounced, suggesting enhanced local structural coherence. For the *x* = 0.22 composition, the local lattice contrast appears more spatially continuous, with fewer regions showing less coherent lattice contrast. Selected area electron diffraction (SAED) and nanobeam electron diffraction (NBED) were performed along the [211] zone axis, which show fundamental perovskite reflections, without clear superlattice spots, diffuse scattering, or spot splitting (Supplementary Fig. 11). Thus, the results cannot directly support a definitive assignment of local order symmetry, possibly due to the limited sensitivity of the technique. To further investigate the nanoscale structures, STEM-EDS mapping was performed for *x* = 0.16 (Supplementary Fig. 12). The HAADF-STEM images reveal nanoscale contrast fluctuations, indicating local structural heterogeneity within the matrix. Corresponding elemental maps show homogeneous distribution throughout the scanned area. These observations are consistent with hierarchical, continuous and overlapping local polar correlations, whose strength, spatial coherence and reversibility evolve progressively with Ti content, consistent with the Raman and PFM findings.

Using qualitative first principles, local approximant calculations and composition-dependent electrical and structural characterization, we explored the evolution of local polarization tendencies and functional responses in the Sm-doped PMN-PT system. The selected DFT configurations qualitatively suggest composition-dependent differences in local polarization tendency, including 〈110〉-like, multidirectional states and 〈001〉-like orientations across the studied compositions. Systematic low-level Ti substitution in Sm_0.025_Pb_0.9625_(Mg_1/3_Nb_2/3_)_1-*x*_Ti_*x*_O_3_ ceramics was investigated to establish a composition-dependent evolution of dielectric, ferroelectric and electromechanical responses in a single material platform. Despite the average cubic symmetry revealed by XRD, compositions with *x* < 0.16 exhibit highly dynamic polar correlations, whose local polar coherence and field responsiveness are enhanced with Ti substitution, enabling rapid and reversible dipolar reorientation under electric bias. Near the relaxor response window, the *x* = 0.16 composition shows an optimized balance between developed local polar response and limited remanent polar stabilization. This material yields a reversible dielectric tunability of 76.4% and *ca*. 0.31% strain (corresponding to *d*_33_^*^ of 1550 pm V^−1^) under 2 kV mm^−1^ with low remanent polarization and low irreversible tunability. In contrast, higher Ti contents (*x* > 0.16) promote stronger and more stable local polar correlations, resulting in increased remanent polarization and a larger irreversible tunability component. The composition with *x* = 0.22 achieves a high strain of 0.54% at 2 kV mm^−1^ (corresponding to *d*_33_^*^ of 2700 pm V^−1^). This high electromechanical response could be attributed to stronger field-induced polar reorientation and increased stability of local polar coherence. These findings demonstrate that composition-driven modulation of local polar correlations near the relaxor response window can enable a practical balance of dielectric agility and large electromechanical strain within a single perovskite platform.

## Methods

### Sample preparation

Compositions with the general formula Sm_0.025_Pb_0.9625_(Mg_1/3_Nb_2/3_)_1-*x*_Ti_*x*_O_3_ (SPMNT, *x* = 0.08, 0.10, 0.12, 0.14, 0.16, 0.18, 0.20 and 0.22) were synthesized via a two-step solid-state reaction method^44^. In the first step, stoichiometric amounts of magnesium oxide (MgO, 99%, Sigma Aldrich) and niobium oxide (Nb_2_O_5_, 99.9%, Sigma Aldrich) were ball-milled in ethanol at 180 rpm for 20 h using a planetary ball mill (Fritsch Pulverisette 5/4 classic line, Germany) with zirconia balls. The dried slurry was then calcined at 1200 °C for 2 h to obtain MgNb_2_O_6_. In the second step, the resulting MgNb_2_O_6_ was mixed with samarium oxide (Sm_2_O_3_, 99.9%, Sigma Aldrich), lead oxide (Pb_3_O_4_, 99%, Sigma Aldrich) and titanium oxide (TiO_2_, 99.8%, Sigma Aldrich) and ball milled again in ethanol at 180 rpm for 20 h. The mixture was dried, sieved through a 250 µm mesh and calcined at 840 °C for 4 h. After cooling, the calcined powder was ball-milled once more, dried and sieved. The resulting fine powder was then uniaxially pressed into 13 mm diameter pellets of *ca*. 1–2 mm thickness at 150 MPa. Finally, the pellets were sintered at 1200 °C for 2 h. Parallel syntheses were carried out and confirmed the reproducibility of the results. For comparison, undoped PMNT08, PMNT16, PMNT22 and PMNT16v compositions were prepared using the same two-step solid-state reaction and sintering procedure. The density of final pellets was measured by the Archimedes method.

### Material characterization

X-ray powder diffraction (XRD) data were collected using a PANalytical X’Pert Pro diffractometer equipped with an X’Celerator detector and Ni-filtered Cu-Kα radiation (λ = 1.5418 Å). Scans were performed over the 2*θ* range 5–120°, with a step size of 0.00334° and an effective count time of 50 s per step. Rietveld refinements were carried out using the GSAS suite of programs^45^, with a cubic (space group *Pm*−3*m*) or an orthorhombic (space group *Amm*2) perovskite model. The microstructure of sintered ceramics was investigated using scanning electron microscopy (SEM, FEI Inspect-F, Oxford Instruments). Piezoresponse force microscopy (PFM) was conducted using a Bruker Dimension Icon AFM system (USA) with an SCM-PIT-V2 conductive probe (Bruker, USA). Raman spectroscopy was performed at room temperature on ceramic samples using a Renishaw inVia^TM^ confocal Raman microscope equipped with a 532 nm excitation laser. For transmission electron microscopy (TEM), ceramic samples were mechanically thinned to *ca*. 100 μm and subsequently ion-milled to electron transparency. Local structural analysis of compositions with *x* = 0.08, 0.16 and 0.22 was conducted using a JEM-F200 TEM instrument (JEOL, Japan).

### Electrical property measurement

For electrical measurements, sintered pellets were ground to rectangular plates of approximate dimensions 4 × 4 mm^2^ and a thickness of 0.30 ± 0.01 mm and silver paste (Gwent Electronic Materials Ltd., Pontypool, UK) was applied to the parallel faces of ceramic pellets, which were then heated to 450 °C for 10 min to form the electrodes. Dielectric measurements at room temperature were conducted using a precision impedance analyser (Agilent 4294 A), while temperature-dependent measurements were performed using an LCR meter (Agilent 4284 A) connected to a temperature-controlled chamber. Dielectric tunability was measured using an Agilent 4294 A impedance analyser with an AC test voltage of 1 V at 100 kHz under DC bias. The DC field was increased stepwise from zero to the maximum bias and subsequently decreased to zero. At each DC bias step, the field was held for 30 s before recording the capacitance. After unloading, the sample was held at zero bias for 60 s before the recovered zero bias capacitance was measured. The ceramic sample was placed between a spherical top electrode and a flat bottom electrode. The strain was calculated from the measured displacement normalized by the sample thickness. Ferroelectric properties, including current-electric field (*I–E*), polarization-electric field (*P*–*E*) and strain-electric field (*S*–*E*) loops were measured using a ferroelectric hysteresis tester (NPL, UK) and an AC triangular waveform at 1 Hz. The piezoelectric coefficient (*d*_33_) was measured using a quasi-static *d*_33_ meter (ZJ-3B Institute of Acoustics, Academia Sinica, China). Current density (*J*) was measured at room temperature under a DC electric field of 2 kV mm^−1^. The field was applied and held for 50 s before recording the current density. The *P–E* and *I–E* loops were measured separately using a ferroelectric hysteresis tester at 1 Hz. No explicit leakage subtraction was applied to the *P–E* loops.

The total dielectric tunability (*η*_total_) was calculated as

1 $${\eta }_{{total}}=\frac{{\varepsilon {\prime} }_{0}-{\varepsilon {\prime} }_{E}}{{\varepsilon {\prime} }_{0}}$$

where ε'_0_ and ε'_*E*_ were the dielectric permittivity at zero and under an applied DC electric field, respectively. After removal of the DC field, the zero bias permittivity was remeasured as ε'_recovered_. The irreversible component (*η*_irreversible_) was calculated as

2 $${\eta }_{{irreversible}}=\frac{{\varepsilon {\prime} }_{0}-{\varepsilon {\prime} }_{{{{\rm{recovered}}}}}}{{\varepsilon {\prime} }_{0}}$$

and the reversible component (*η*_reversible_) was obtained as

3 $${\eta }_{{reversible}}={\eta }_{{total}}-{\eta }_{{irreversible}}$$

### DFT simulation

First principles density functional theory (DFT) calculations were performed using Vienna Ab initio Simulation Package (VASP)^46^, employing the projector augmented-wave (PAW)^47^ method with Perdew-Burke-Ernzerhof (PBE)-based^48^ pseudopotentials. The r^2^SCAN meta-GGA functional was used to capture exchange-correlation effects^49^. To investigate the effect of Ti substitution, simplified 3 × 3 × 3 local approximant supercells were constructed using XRD-refined cell parameters. The selected supercells, with full formulas of SmPb_26_Mg_8_Nb_17_Ti_2_O_81_, SmPb_26_Mg_8_Nb_15_Ti_4_O_81_, SmPb_26_Mg_7_Nb_14_Ti_6_O_81_ and Pb_27_Mg_8_Nb_17_Ti_2_O_81_, were used as representative local approximants to the nominal *x* = 0.08, 0.16, 0.22 compositions and undoped PMNT08, respectively. The B-site occupancies were chosen to match the target integer Mg/Nb/Ti stoichiometries allowed by the supercell. To minimize artificial Ti clustering, Ti atoms were deliberately distributed over both edge and center B sites within the supercell. For the *x* = 0.16 composition, seven additional configurations were generated by varying the Mg/Nb/Ti distribution within the same supercell composition and then relaxing the atomic positions under the same fixed lattice parameters. The simplified Sm-containing supercells contain one Sm atom per 27 A-sites and do not explicitly include A-site vacancies; therefore, they are treated as selected local approximant models of possible chemical environments. A plane-wave cutoff energy of 600 eV and a Monkhorst-Pack 3 × 3 × 3 k-point mesh were used. The electronic convergence criterion was set to 10^−6^ eV, with Gaussian smearing (ISMEAR = 0, σ = 0.05 eV). The DFT + U method (Dudarev scheme, LDAUTYPE = 2)^50^, was applied to correct on-site Coulomb interactions for the transition metals *d* orbitals, with U = 1.5 eV for Nb 4*d* and U = 2.0 eV for Ti 3*d* states. Atomic positions were relaxed using the conjugate gradient algorithm until the Hellmann-Feynman forces were below 0.02 eV Å^−1^, while keeping cell parameters fixed. Following full relaxation, density functional perturbation theory (DFPT) calculations were conducted to compute the Born effective charge (|Z^*^|) via the linear response method, using a tightened electronic energy convergence criterion of 10^−8^ eV. The spontaneous polarization components (P_x_, P_y_ and P_z_) were calculated by using the Born effective charges and atomic displacements relative to the initial centrosymmetric structure. The resulting Born effective charges and relaxed atomic positions were subsequently used to calculate spontaneous polarization. The magnitude of net polarization vector |P| was calculated via:

4 $$\left|P\right|=\sqrt{{P}_{x}^{2}+{P}_{y}^{2}+{P}_{z}^{2}}$$

The corresponding polarization direction unit vectors were determined as:

5 $${u}_{x}=\frac{{P}_{x}}{\left|P\right|},\,{u}_{y}=\frac{{P}_{y}}{\left|P\right|},\,{u}_{z}=\frac{{P}_{z}}{\left|P\right|}$$

### Reporting summary

Further information on research design is available in the Nature Portfolio Reporting Summary linked to this article.

## Supplementary information

Supplementary Information

Reporting Summary

Transparent Peer Review file

## Source data

Source Data

## Data Availability

All data needed to evaluate the conclusions in the paper are present in the paper and/or the Supplementary Materials. Source Data are provided with this paper, including DFT structures. Any other relevant data is also available from the corresponding authors upon request. Source data are provided with this paper.
