# Supplementary material for: Large strain and high reversible dielectric tunability in Sm-modified PMN-PT ceramics
Source: Nat Commun. 2026 Sep 18;17:10941. doi: 10.1038/s41467-026-77914-1 (PMC13680613; doi:10.1038/s41467-026-77914-1)
Supplement: Supplementary file 1 [file 41467_2026_77914_MOESM1_ESM.pdf]

Supplementary Materials for

Large strain and high reversible dielectric tunability in Sm-modified PMN-PT ceramics

Hangfeng Zhang,[1] Theo Graves Saunders,[1] Haixue Yan[2]*, Yang Hao[1]*

Affiliations:

[1]School of Electronic Engineering and Computer Science, Queen Mary University of London, Mile End Road London E1 4NS, UK

[2]School of Engineering and Materials Science, Queen Mary University of London, Mile End Road London E1 4NS, UK

*Corresponding authors' Emails: h.x.yan@qmul.ac.uk and y.hao@qmul.ac.uk.

The PDF file includes:

Figs. S1 to S12
Table S1

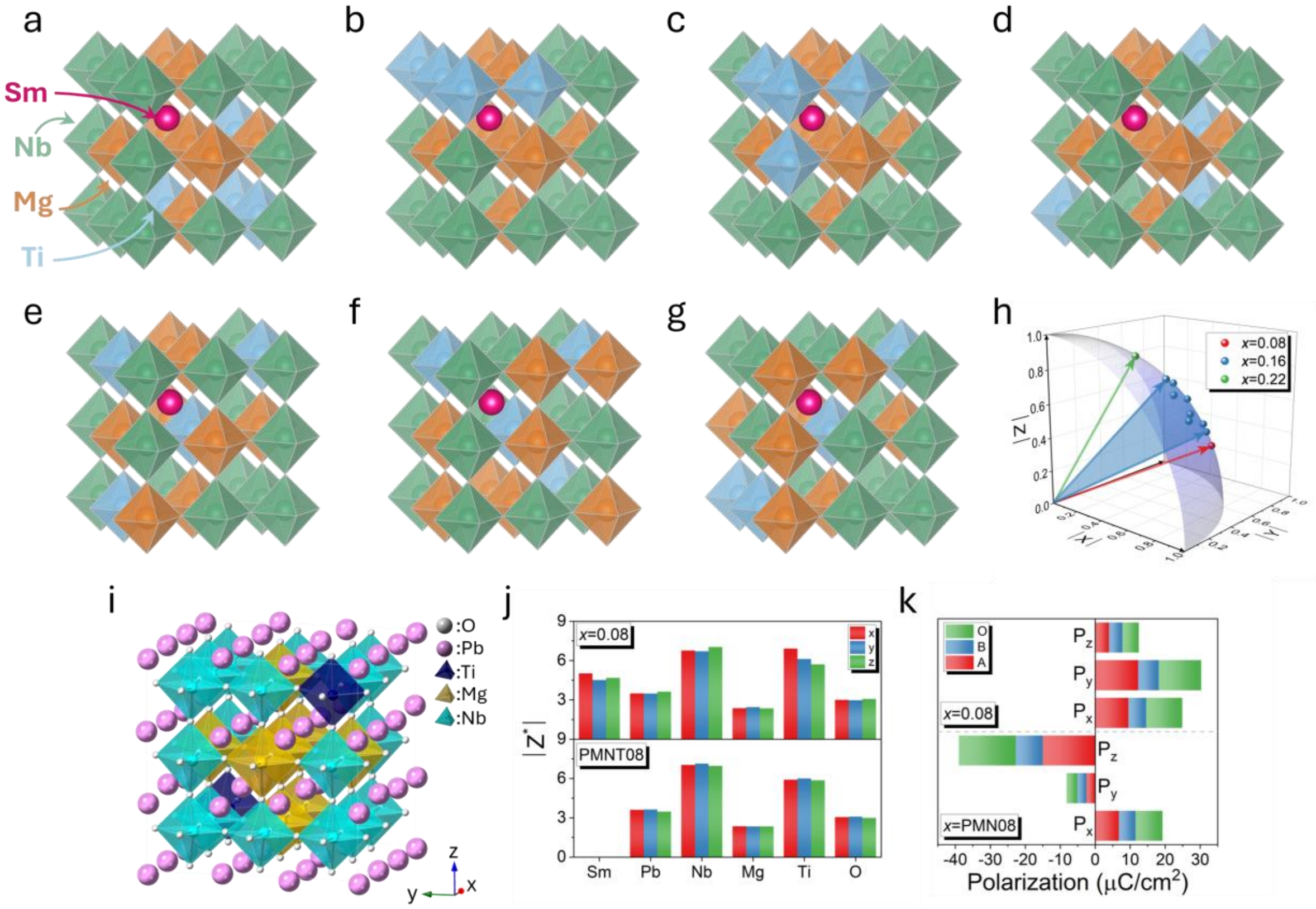

Supplementary Fig. 1. (a-g) Representative structures of seven additional x = 0.16 configurations; (h) Normalized polarization directions obtained from relaxed DFT supercells for x = 0.08, x = 0.16 and x = 0.22; (i) DFT-relaxed crystal structure for PMNT08 composition. (j) Born effective charge of each element and (k) net polarization along each crystallographic axis for PMNT08 and x = 0.08 compositions.

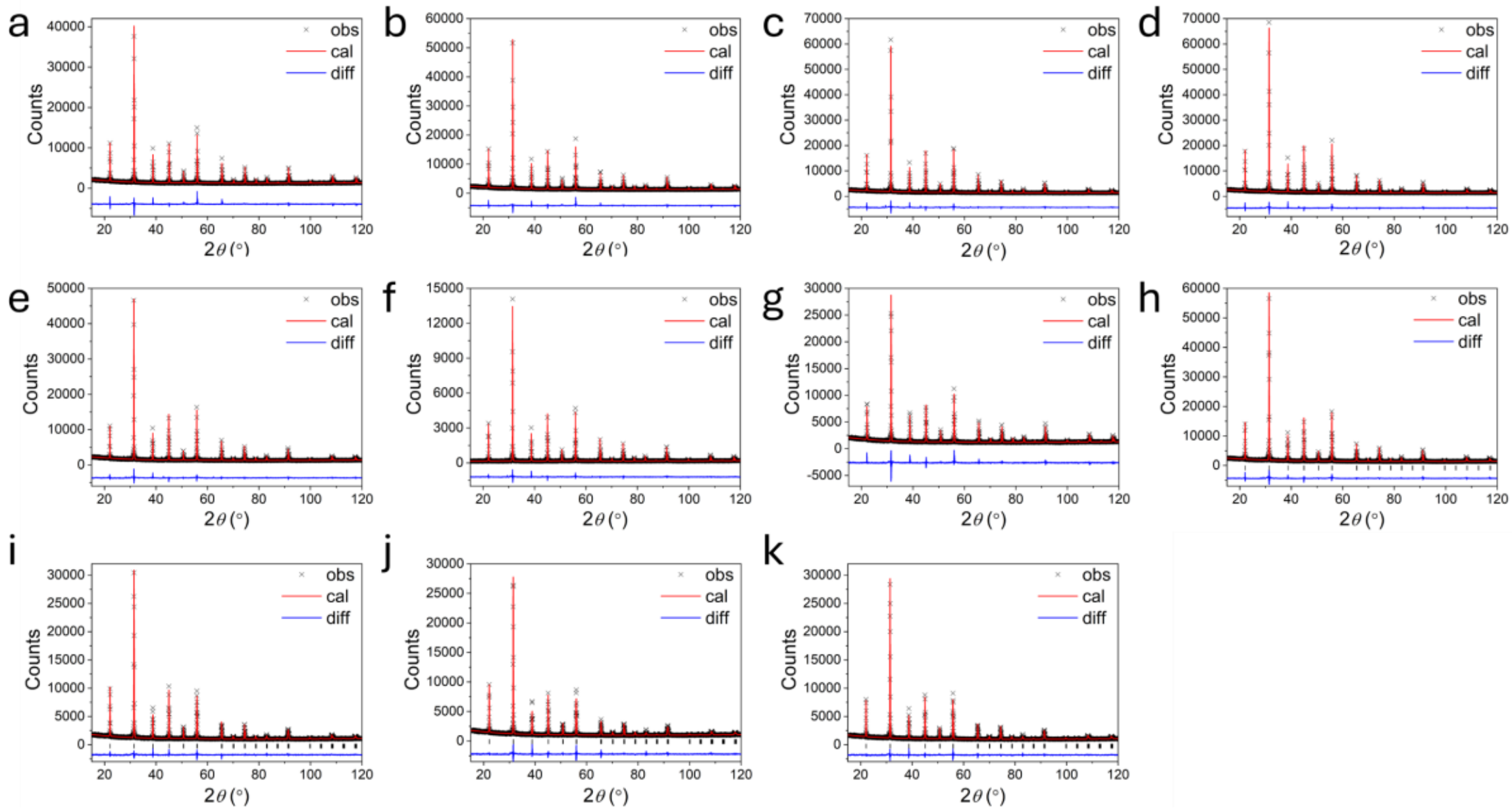

Supplementary Fig. 2. Fitted XRD profiles for SmPMNT and undoped PMNT compositions, (a) $x = 0.08$, (b) $x = 0.10$, (c) $x = 0.12$, (d) $x = 0.14$, (e) $x = 0.18$, (f) $x = 0.20$ and (g) $x = 0.22$, and PMN-xPT: (h) PMNT08, (i) PMNT16, (j) PMNT22 and (k) PMNT16v.

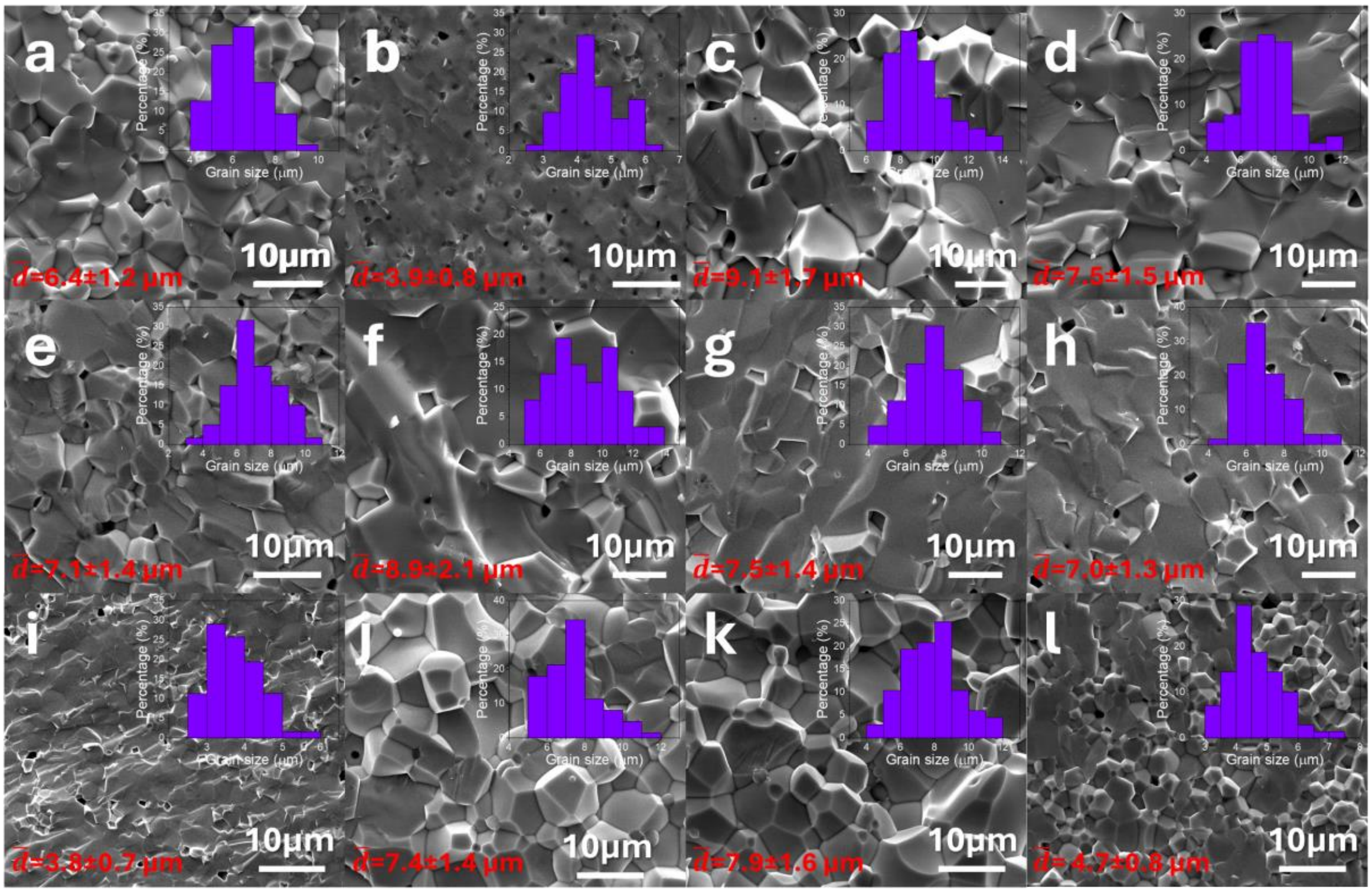

Supplementary Fig. 3. SEM images of cross-section for SmPMNT compositions, (a) $x = 0.08$, (b) $x = 0.10$, (c) $x = 0.12$, (d) $x = 0.14$, (e) $x = 0.16$, (f) $x = 0.18$ (g) $x = 0.20$, (h) $x = 0.22$, PMN-xPT: (i) PMNT08, (j) PMNT16, (k) PMNT22 and (l) PMNT16v with average grain size ($\bar{d}$).

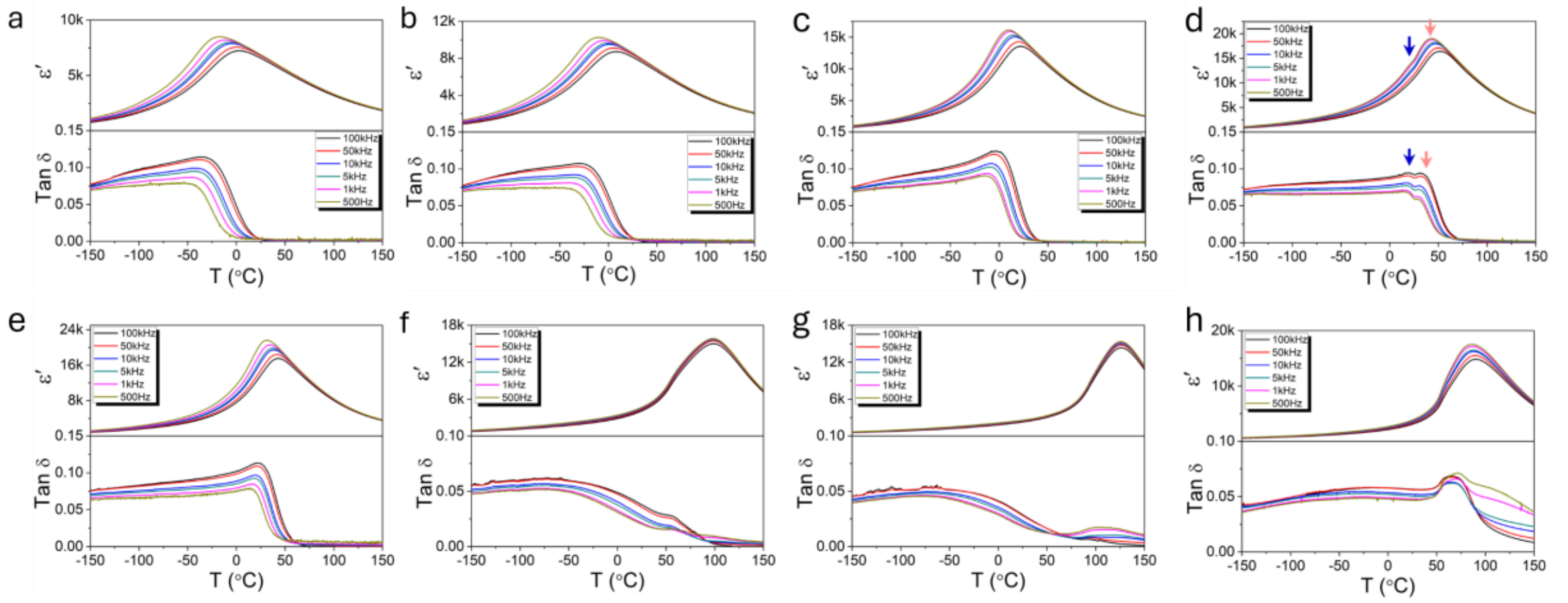

Supplementary Fig. 4. Temperature dependence of dielectric permittivity and loss for SmPMNT and undoped PMNT compositions, (a) $x = 0.10$, (b) $x = 0.12$, (c) $x = 0.14$, and (d) $x = 0.20$, (e) PMNT08, (f) PMNT16, (g) PMNT22 and (h) PMNT16v.

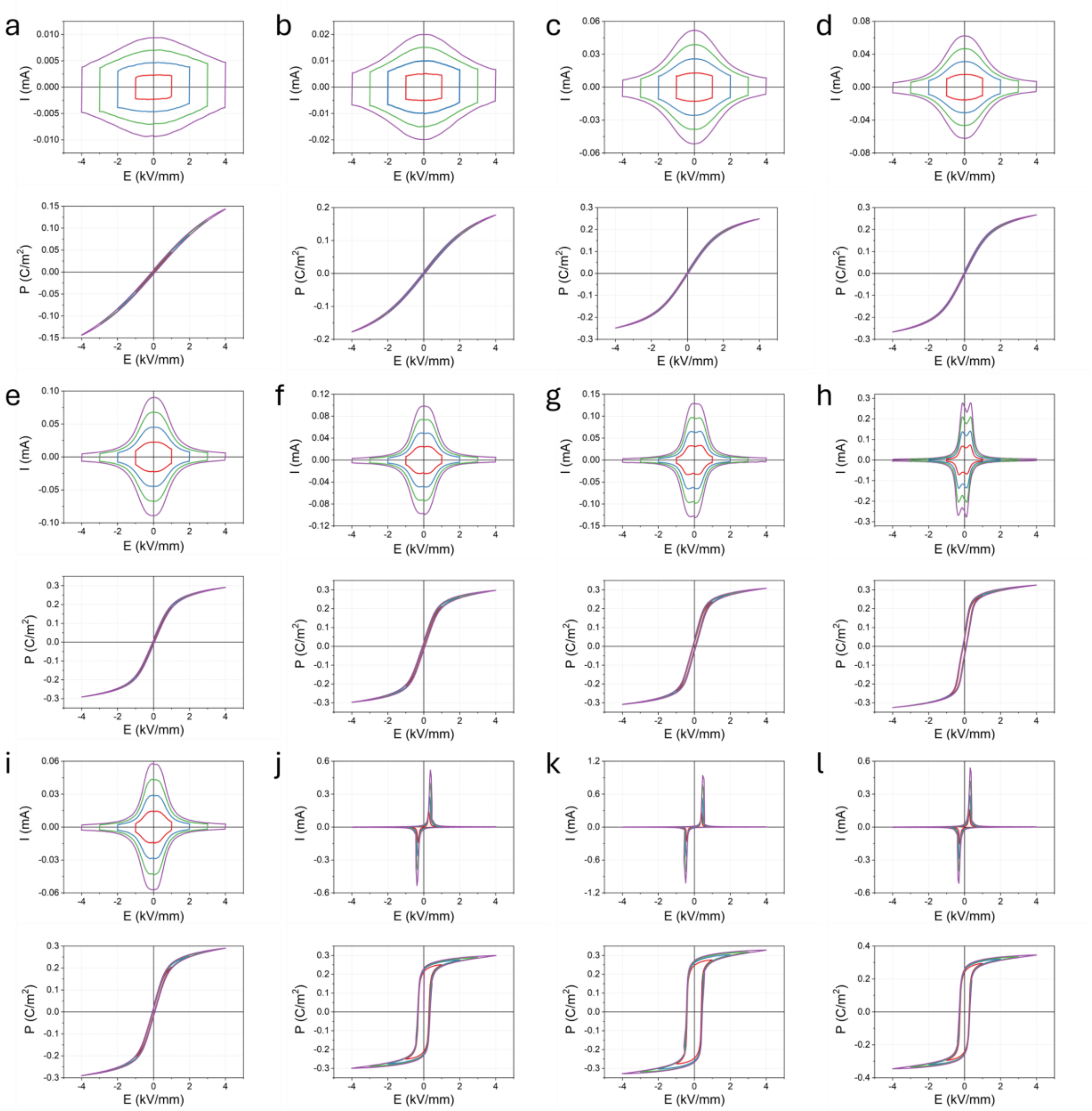

Supplementary Fig. 5. *P-E* and *I-E* loop profiles for SmPMNT and undoped PMNT compositions measured at 1 Hz. (a) x = 0.08, (b) x = 0.10, (c) x = 0.12, (d) x = 0.14, (e) x = 0.16 (f) x = 0.18, (g) x = 0.20, (h) x = 0.22, (i)PMNT08, (j) PMNT16, (k) PMNT22 and (l) PMNT16v.

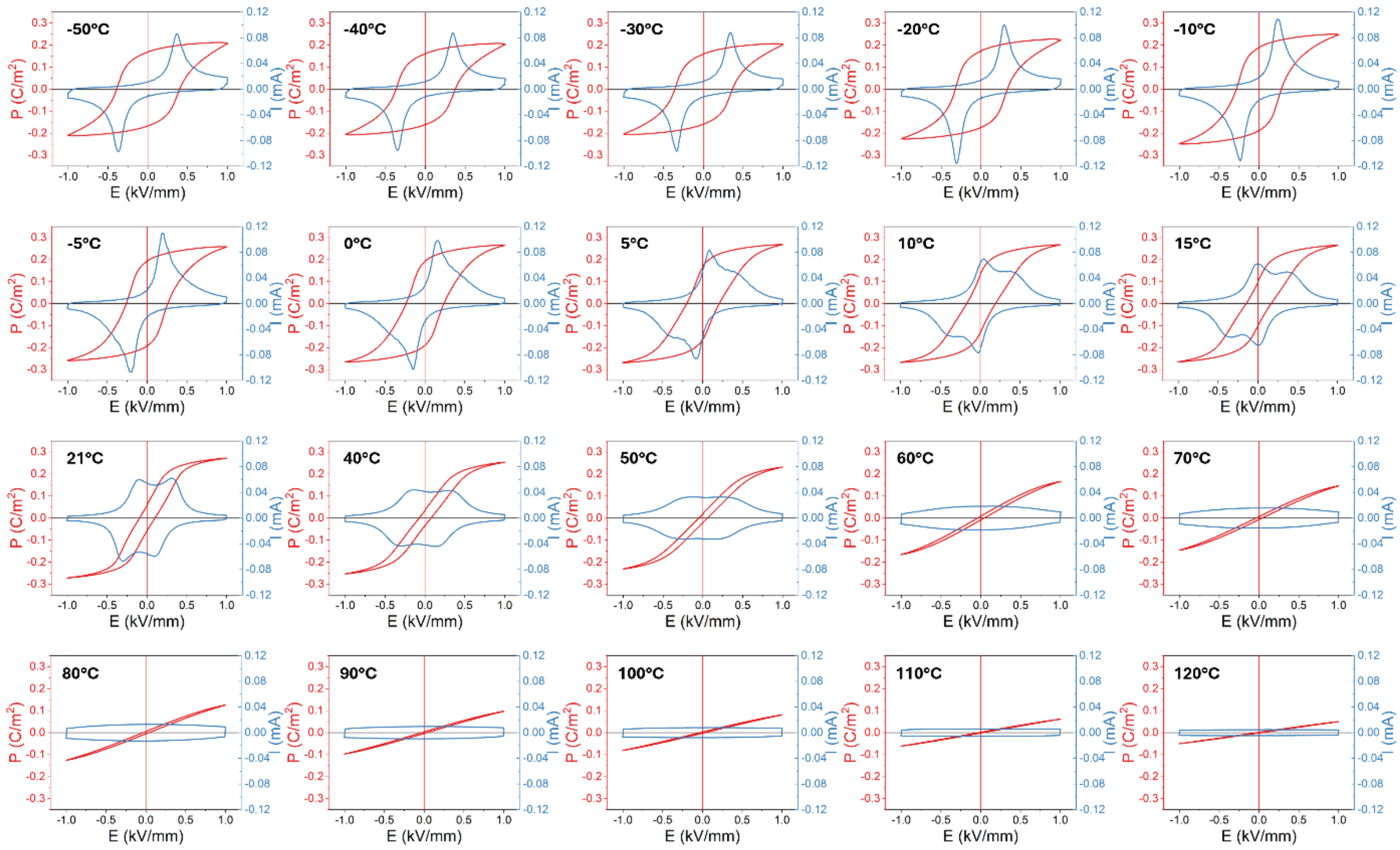

Supplementary Fig. 6. Temperature dependence of *P-E* and *I-E* loops for x = 0.22 composition measured at 1 Hz.

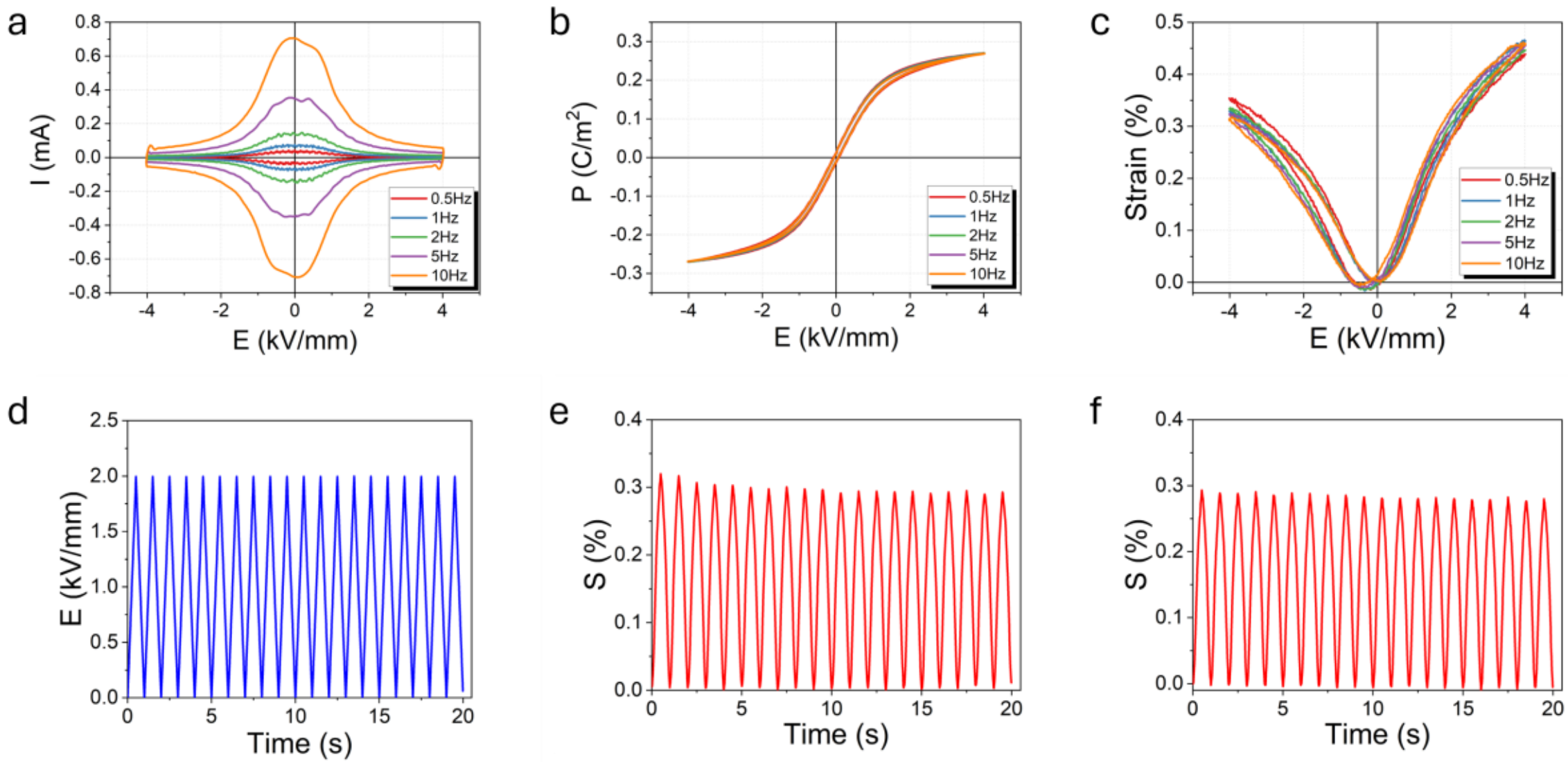

Supplementary Fig. 7. Frequency dependence of (a) *I*-*E*, (b) *P*-*E* and (c) S-*E* loops for x = 0.16 composition. Electric field induced cyclic strain of the x = 0.16 composition: (d) applied cyclic electric field, (e) strain response of the fresh sample and (f) strain response after 100 cycles.

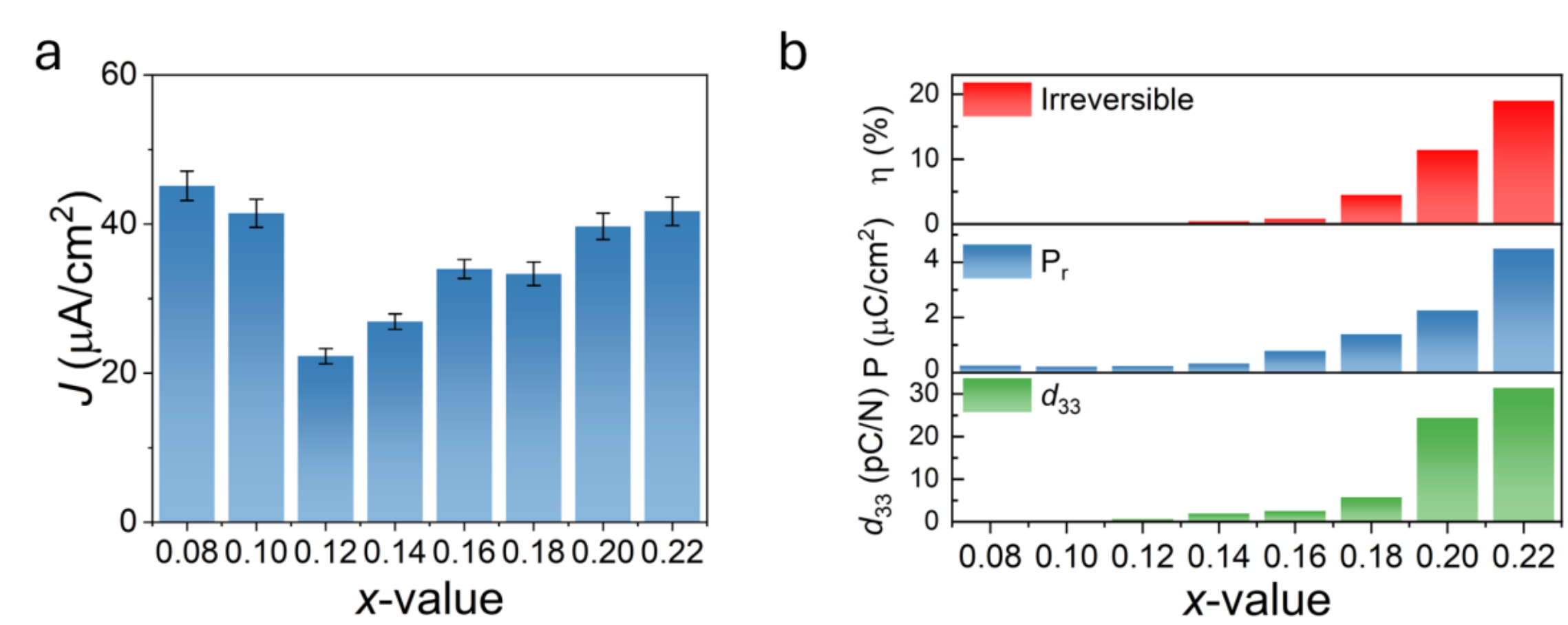

Supplementary Fig. 8. (a) Current density (J) of the studied SPMNT compositions under a 2 kV mm^{-1} DC electric field. Error bars represent the calculated uncertainty of the derived value; (b) Composition dependence of irreversible tunability, remanent polarization (P_r) and small-signal piezoelectric coefficient (d_{33}) in the SPMNT system.

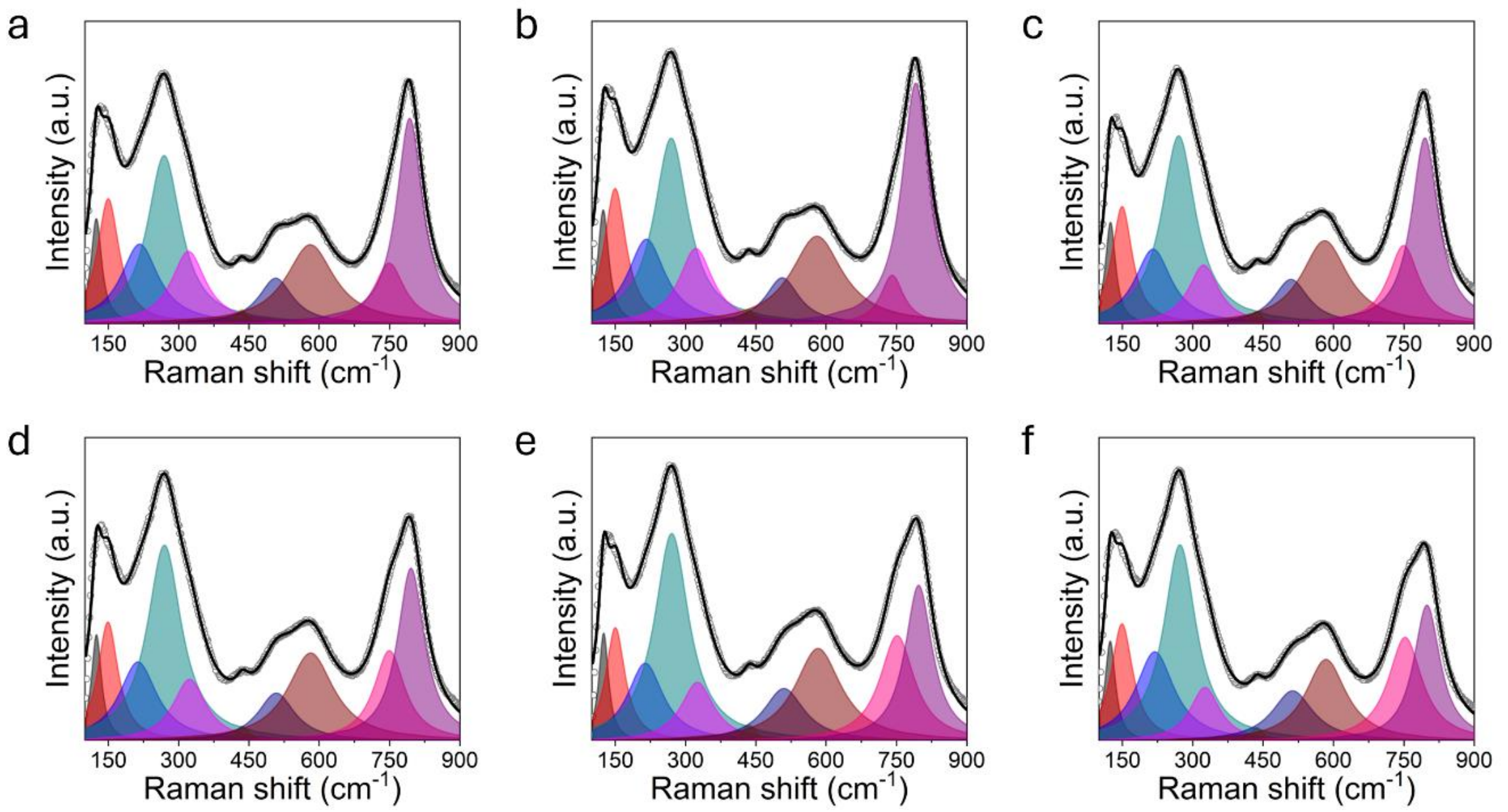

Supplementary Fig. 9. Fitted Raman spectra for (a) x = 0.12, (b) x = 0.14, (c) x = 0.16, (d) x = 0.18, (e) x = 0.20 and (f) x = 0.22.

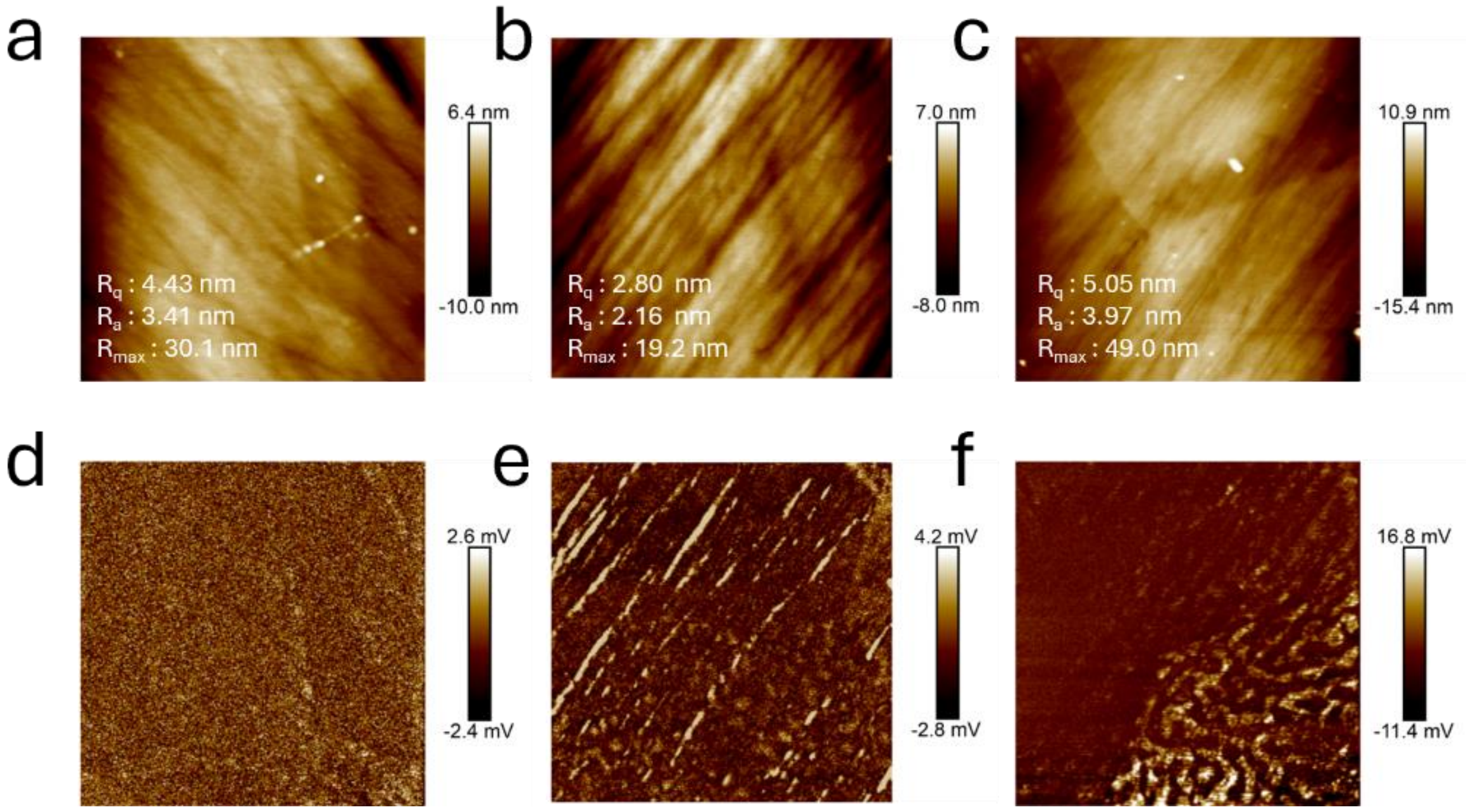

Supplementary Fig. 10. PFM (a-c) topography and (d-f) amplitude images for (a, d) x = 0.08, (b, e) x = 0.16 and (c, f) x = 0.22 compositions.

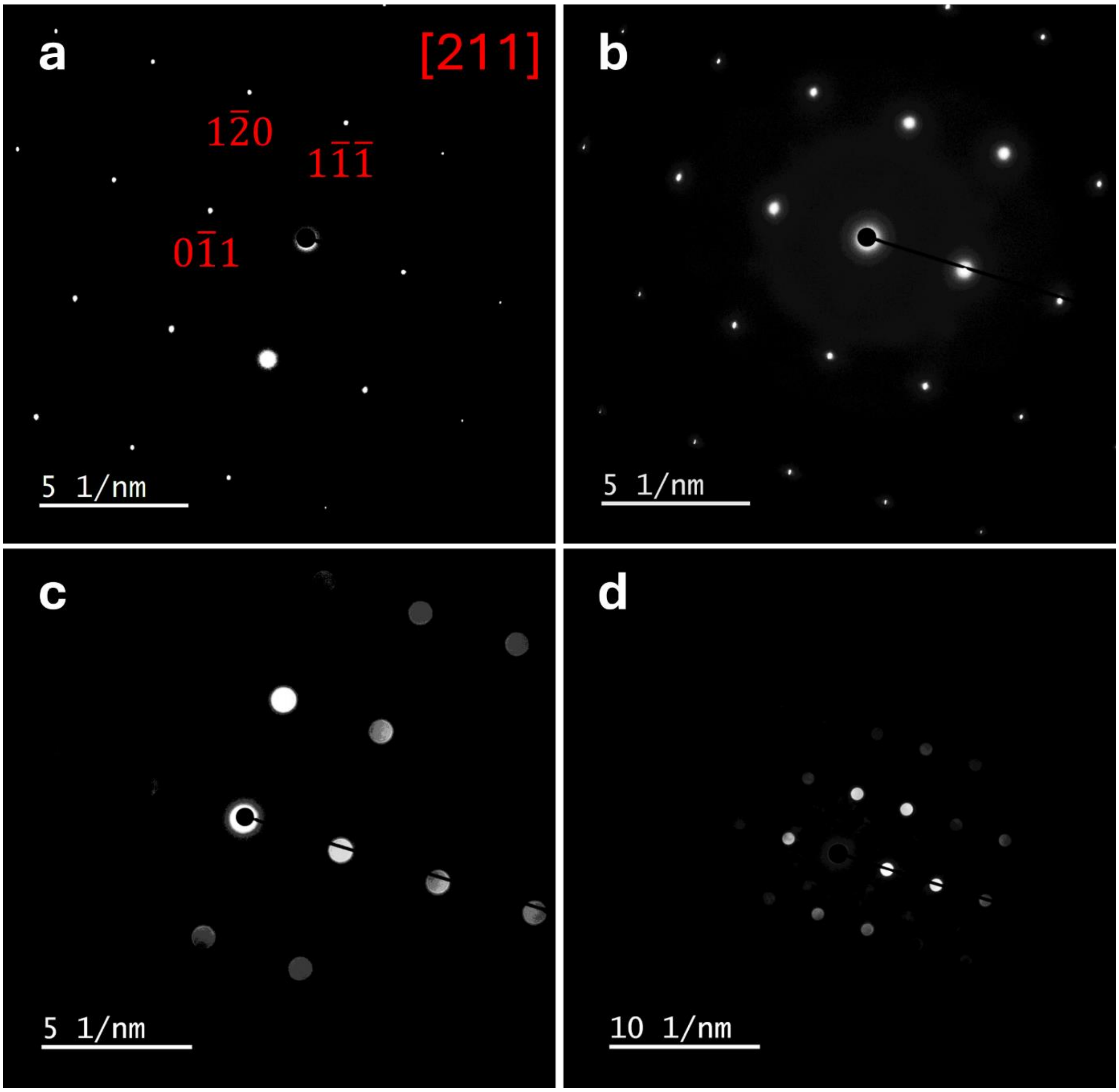

Supplementary Fig. 11. Electron diffraction patterns obtained from the x = 0.16 ceramic along the [211] zone axis. (a) Selected-area electron diffraction (SAED) pattern showing the average crystallographic structure. (b-d) Nanobeam electron diffraction (NBED) patterns collected from different local regions.

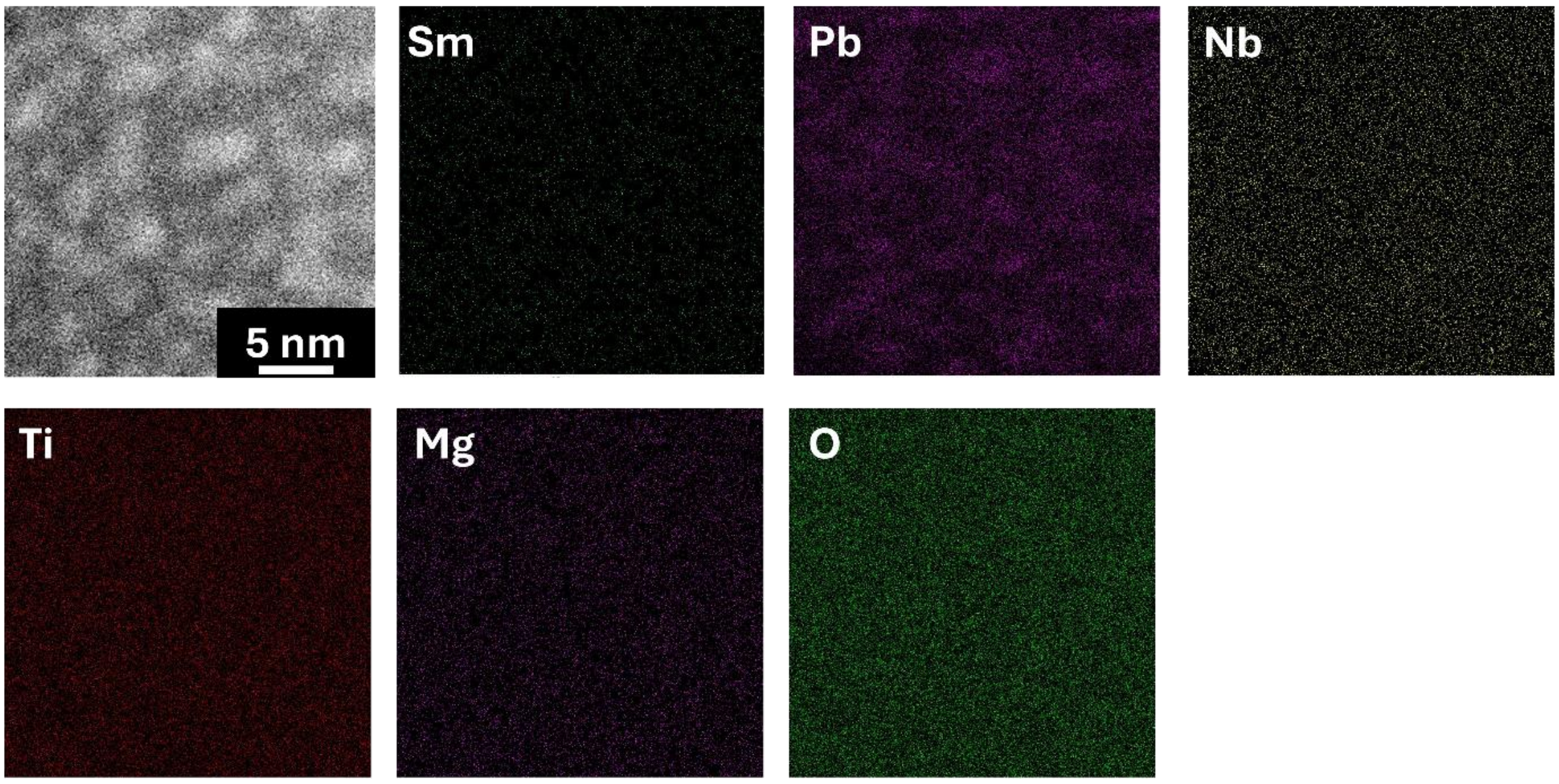

Supplementary Fig. 12. STEM-HAADF images and corresponding EDS elemental mapping of the $x = 0.16$ composition.

Supplementary Table 1. Crystal and refinement parameters for fits to studied compositions. Estimated standard deviations are given in parentheses.

Chemical formula	$Sm_{0.025}Pb_{0.9625}(Mg_{1/3}Nb_{2/3})_{0.92}Ti_{0.08}O_3$	$Sm_{0.025}Pb_{0.9625}(Mg_{1/3}Nb_{2/3})_{0.90}Ti_{0.10}O_3$	$Sm_{0.025}Pb_{0.9625}(Mg_{1/3}Nb_{2/3})_{0.88}Ti_{0.12}O_3$	$Sm_{0.025}Pb_{0.9625}(Mg_{1/3}Nb_{2/3})_{0.86}Ti_{0.14}O_3$
**Space group**	*Pm*-3*m*	*Pm*-3*m*	*Pm*-3*m*	*Pm*-3*m*
**Unit cell dimensions**	*a* = 4.03579(3) Å	*a* = 4.03444(2) Å	*a* = 4.03333(5) Å	*a* = 4.03144(3) Å
**Volume (Å^3)**	65.734(1)	65.667(1)	65.613(3)	65.521(2)
**Z**	1	1	1	1
**D_{calc} (g cm^{-3})**	8.070	8.067	8.062	8.062
**R-factors**	R_{wp} = 0.0444<br>R_p = 0.0327<br>χ^2 = 3.699<br>R_F^2 = 0.0517<br>R_{exp} = 0.0232	R_{wp} = 0.0448<br>R_p = 0.0331<br>χ^2 = 3.836<br>R_F^2 = 0.0484<br>R_{exp} = 0.0229	R_{wp} = 0.1162<br>R_p = 0.0882<br>χ^2 = 2.914<br>R_F^2 = 0.0597<br>R_{exp} = 0.0677	R_{wp} = 0.0984<br>R_p = 0.0759<br>χ^2 = 2.176<br>R_F^2 = 0.0598<br>R_{exp} = 0.0669
**No. of variables**	22	22	22	22
**No. of profile points**	3649	3649	3440	3440

Chemical formula	$Sm_{0.025}Pb_{0.9625}(Mg_{1/3}Nb_{2/3})_{0.84}Ti_{0.16}O_3$	$Sm_{0.025}Pb_{0.9625}(Mg_{1/3}Nb_{2/3})_{0.82}Ti_{0.18}O_3$	$Sm_{0.025}Pb_{0.9625}(Mg_{1/3}Nb_{2/3})_{0.80}Ti_{0.20}O_3$	$Sm_{0.025}Pb_{0.9625}(Mg_{1/3}Nb_{2/3})_{0.78}Ti_{0.22}O_3$
**Space group**	*Pm*-3*m*	*Pm*-3*m*	*Pm*-3*m*	*Pm*-3*m*
**Unit cell dimensions**	*a* = 4.02954(3) Å	*a* = 4.02852(4) Å	*a* = 4.02654(5) Å	*a* = 4.02461(2) Å
**Volume (Å^3)**	65.429(2)	65.379(2)	65.283(1)	65.189(1)
**Z**	1	1	1	1
**D_{calc} (g cm^{-3})**	8.063	8.046	8.058	8.058
**R-factors**	R_{wp} = 0.0993<br>R_p = 0.0760<br>χ^2 = 2.732<br>R_F^2 = 0.0622<br>R_{exp} = 0.0603	R_{wp} = 0.0511<br>R_p = 0.0358<br>χ^2 = 4.078<br>R_F^2 = 0.0874<br>R_{exp} = 0.0254	R_{wp} = 0.0482<br>R_p = 0.0342<br>χ^2 = 3.767<br>R_F^2 = 0.0848<br>R_{exp} = 0.0249	R_{wp} = 0.0476<br>R_p = 0.0345<br>χ^2 = 3.966<br>R_F^2 = 0.0873<br>R_{exp} = 0.0240
**No. of variables**	22	18	20	22
**No. of profile points**	3440	3649	3649	3649

Chemical formula	$Pb(Mg_{1/3}Nb_{2/3})_{0.92}Ti_{0.08}O_3$	$Pb(Mg_{1/3}Nb_{2/3})_{0.84}Ti_{0.16}O_3$	$Pb(Mg_{1/3}Nb_{2/3})_{0.78}Ti_{0.22}O_3$	$Pb_{0.9875}(Mg_{1/3}Nb_{2/3})_{0.84}Ti_{0.16}O_3$
**Space group**	*Pm*-3*m*	*Amm*2	*Amm*2	*Amm*2
**Unit cell dimensions**	*a* = 4.03896(3) Å	*a* = 4.0340(1) Å<br>*b* = 5.7006(1) Å<br>*c* = 5.7125(1) Å	*a* = 4.0276(2) Å<br>*b* = 5.6914(2) Å<br>*c* = 5.7064(2) Å	*a* = 4.0335(1) Å<br>*b* = 5.7004(2) Å<br>*c* = 5.7119(2) Å
**Volume (Å^3)**	65.889(2)	131.369(7)	130.81(1)	131.337(9)
**Z**	1	2	2	2
**D_{calc} (g cm^{-3})**	8.152	8.133	8.134	8.069
**R-factors**	R_{wp} = 0.0455<br>R_p = 0.0341<br>χ^2 = 3.944<br>R_{F^2} = 0.0619<br>R_{exp} = 0.0230	R_{wp} = 0.0429<br>R_p = 0.0328<br>χ^2 = 2.500<br>R_{F^2} = 0.0761<br>R_{exp} = 0.0273	R_{wp} = 0.0491<br>R_p = 0.0354<br>χ^2 = 3.196<br>R_{F^2} = 0.0812<br>R_{exp} = 0.0276	R_{wp} = 0.0421<br>R_p = 0.0318<br>χ^2 = 2.308<br>R_{F^2} = 0.0717<br>R_{exp} = 0.0278
**No. of variables**	22	26	26	26
**No. of profile points**	3649	3649	3649	3649
