# Supplementary material for: Large strain and high reversible dielectric tunability in Sm-modified PMN-PT ceramics
Source: Nat Commun. 2026 Sep 18;17:10941. doi: 10.1038/s41467-026-77914-1 (PMC13680613; doi:10.1038/s41467-026-77914-1)
Supplement: Supplementary file 3 [file 41467_2026_77914_MOESM3_ESM.pdf]

nature portfolio

Peer Review File

Large strain and high reversible dielectric tunability in Sm-modified PMN-PT ceramics

Corresponding Author: Professor Yang Hao

This file contains all reviewer reports in order by version, followed by all author rebuttals in order by version.

Version 0:

Reviewer comments:

Reviewer #1

(Remarks to the Author)
In this work, the authors relatively large strain with low hysteresis in Sm-doped PMN-PT perovskite relaxor ferroelectric ceramics through polar nanoregion engineering. Specifically, the authors first calculated the impacts of Sm and Ti on the free energy landscape of PMN, then they designed a series of PMN-based ceramics with interesting electrostrictive and dielectric properties. Overall, I think this manuscript is worth for publication in Nature Communications as the authors addressed the following comments.
(1) For DFT calculations, the impact of Sm should be further discussed, e.g., its impact on polarization magnitude and orientation. Currently, only the concentration of Ti was emphasized.

(2) The difference between calculation and experimental results is quite large for the PMN-based materials. It is understandable that the temperature and crystallographic orientations are different for calculations and experiments. However, how the authors use the calculation predicted data assists the design of real materials should be discussed.

(3) The strain of PMN-0.22PT composition is quite large in polycrystalline ceramics. This point should be further discussed and highlighted in the abstract and conclusion.

(4) The relationship between electrostrictive strain and dielectric tunnability isn't clear. The question here is that why the present work focuses on these two characteristics of PMN system.

(5) The quality of TEM images isn't high. I don't think the marked regions are chemical ordered regions, since there isn't any superlattice diffraction point presented here. If they are not chemical ordered regions, the authors have to indicate what the "ordered" means.

Reviewer #2

(Remarks to the Author)
This manuscript reports a Sm-doped PMN-PT ceramic series and proposes that a composition near x = 0.16 offers a favorable balance of reversible strain, dielectric tunability, and low hysteresis. The topic is potentially interesting, and the search for a composition window that balances multiple functional metrics within one relaxor family is worthwhile.
In the comments below, I assess the manuscript in its present form and base my evaluation on the statements, quantitative assignments, and interpretations as they are currently written, rather than on probable intended meanings where these are not stated clearly.
One of the most important issues is the inconsistency in the strain / d33* assignment. The Abstract and Outlook assign ~0.31% strain at 2 kV mm−1 to x = 0.16, whereas the Results section assigns 0.54% to x = 0.16 and 0.31% to x = 0.22, with the corresponding d33* values shifting accordingly. As written, this creates ambiguity regarding which composition is actually being identified as the optimum. The manuscript would benefit from a careful consistency check across the Abstract, Results, figure discussion, captions, and Outlook.
The study is framed as a Sm-doped PMN-PT design strategy, yet no undoped PMN-PT control series prepared and measured under comparable conditions is presented. As a result, it is difficult to distinguish cleanly among Ti-composition effects, Sm-substitution effects, and processing or microstructural contributions. Including such a control would significantly strengthen the central design argument.

The current computational analysis does not appear sufficient to support the broader structural interpretation advanced in the manuscript. The calculations do not seem to represent the likely Sm-related charge-compensation chemistry, they use very small ordered supercells that do not meaningfully sample relaxor disorder, and they are performed with the lattice fixed to XRD-derived parameters despite the central importance of strain-polarization coupling in this system. Taken together, these limitations weaken strong inferences about polarization rotation, isotropy, or a flattened energy landscape. In its current form, the DFT section seems more suitable for a limited discussion of local polarization tendencies based on small-cell models than for supporting the stronger PNR-scale and crossover claims made in the manuscript.

Language around PNRs should also be made more cautious. The manuscript repeatedly uses wording suggesting that discrete, isolable PNR objects and their density or alignment are being engineered directly. This interpretation seems stronger than the present methods can firmly establish, and it does not engage fully with more recent PMN / PMN-PT literature, which often favors hierarchical polar correlations with smooth spatial variation or continuous and overlapping polarization textures over a simple independent-PNR picture. The argument would be more convincing if it more clearly separated what is directly measured from what is inferred.

The structural interpretation also appears more specific than the data strictly support. All compositions are refined using an average cubic Pm-3m model, yet the manuscript later makes more specific claims regarding local phase competition, increasing ferroelectric order, and domain stabilization. Raman, PFM, and HRTEM may indeed support increasing local polar coherence with Ti content, but they do not seem sufficient, on their own, to establish the full structural specificity of the current narrative. It would therefore be helpful either to soften these interpretations or to provide additional direct symmetry-sensitive evidence.

The electrical evidence would benefit from stronger validation. The key P-E, I-E, and S-E loops are mainly presented at 1 Hz, without a clearly documented study of frequency dependence, leakage contributions, cycling stability, or reproducibility. Because these data are used both to motivate the mechanism and to support application-facing performance claims, a more thorough electrical characterization would strengthen the paper substantially. Relatedly, the pellets are described only as approximately 1-2 mm thick before sintering, while the main figures of merit are expressed in kV mm-1 and d33* = Smax/Emax. Exact final sample thicknesses and field uncertainty are not clearly documented for each measured specimen. Since the central comparison depends directly on electric field, clearer reporting here would be important.

The proposed optimum at x = 0.16 is also not yet established robustly. The paper treats this composition as a special optimum, but that conclusion is not strongly supported statistically. There are no replicate pellets, no uncertainty bars, no batch-to-batch variation, no finer composition spacing near the proposed optimum, and no strain data shown for the compositions x = 0.14 and x = 0.18. At present, the optimum appears suggestive rather than firmly established.

More broadly, the manuscript argues for a special crossover composition near x = 0.16, but it does not yet convincingly show why the observed balance of tunability and strain could not be explained largely as a near-Tm effect within the relaxor response window. In its current form, the paper does not clearly separate a distinct crossover mechanism from a more conventional near-Tm optimization scenario.

There are also several secondary issues that should be corrected carefully. The nominal chemistry is written inconsistently (Nb0.67 versus Nb0.66) across the manuscript and supplement, which is important in a composition-sensitive study. The bibliography appears to contain duplicate entries and at least one incomplete-looking reference('Li et al.' appears as Refs 17 and 29; 'Huangfu et al.' as Refs 20 and 36; the authors own 2025 high-strain paper appears as Refs 30 and 32.). The ionic-radius argument in the unit-cell-volume discussion may need to be revisited, both in terms of coordination-number choice and numerical consistency. Topology images of PFM should be corrected to topography images, and the crystallographic direction notation should be made consistent. The exact tunability protocol details, current normalization, and strain-sensing methodology should also be reported more explicitly.

Reviewer #3

(Remarks to the Author)

The authors developed a polarization landscape engineering strategy to control the anisotropy of PNRs in Sm-doped PMN-PT solid solution by tuning Ti concentration. They achieved 0.31% reversible strain and 77% dielectric tunability under an electric field of 2 kV/mm with minimal hysteresis. The novelty of the current work is not sufficient to warrant the publication in this journal. Besides, the experimental work is not sufficient to support the conclusion.

1. Two lines above Equation (1), the authors claim that the A-site cations and O anions are the main contributors to the spontaneous polarization. Is this conclusion valid for all the different Ti concentrations studied? What is the underlying mechanism that the varied concentration of Ti will affect the polarization direction or the displacement direction of A-site cations? Intuitively, I would expect that as Ti increases in concentration, the off-center displacement direction of Ti cations will be affected.

2. As Ti concentration increases (x>0.16), the authors claim the existence of local orthorhombic and tetragonal phases, which facilitates the easier polarization rotations within PNRs. Is there any experimental evidence for the local orthorhombic and tetragonal phases? Are those local polar phases due to composition inhomogeneities or fluctuations?

3. What is the local symmetry of the PNRs observed in Fig.4? Detailed microstructural characterizations on those PNRs should be conducted, which include diffraction by nano-beam. HAADF-STEM should be conducted to extract the information about the atomic displacement on the region observed.

4. The authors emphasize the minimal hysteresis achieved in x=0.16. However, why do they only report the temperature dependance of the P-E hysteresis loop of the sample at x=0.22 (Fig.3)?

Version 1:

Reviewer comments:

Reviewer #1

(Remarks to the Author)
I'm satisfied with the authors' responses and the corresponding revisions. I don't have further comments on the manuscript.

Reviewer #2

(Remarks to the Author)
I thank the authors for the careful revision and detailed response. The manuscript is clearer, and several additions are genuinely useful: the selected undoped PMN-PT controls, the more cautious DFT framing, the moderated PNR language, the added frequency\cycling data, and the intermediate-composition strain data. I also appreciate that x = 0.16 is now described more carefully as a best-balanced composition within the investigated series, with its proximity to the relaxor response window acknowledged more explicitly.
I want to be clear about what I think is strong here, because it matters for the recommendation. The core functional result, simultaneously achieving sizeable reversible electrostrain, high dielectric tunability, and low remanence/hysteresis in a single bulk ceramic, is a worthwhile and non-trivial achievement, and the composition-resolved dataset is a real contribution. My concern is not the data, but the interpretive framing built on top of it. As written, the paper is presented as a mechanism-resolved demonstration of "PNR engineering" producing an ergodic-nonergodic crossover with a uniquely flattened polarization landscape, and as a route to low-loss multifunctional device performance. The evidence more securely supports a narrower and still valuable message: near-Tm composition-window optimization of strain, dielectric tunability, and low remanence in one Sm/A-site-vacancy-modified PMN-PT ceramic series. The title, abstract and conclusions should be reframed accordingly and should avoid implying a fully mechanism-resolved PNR-engineering strategy, a firmly established ergodic-nonergodic crossover, or low-loss multifunctional device performance.
The proposed mechanism is not yet separated from conventional near-Tm relaxor behavior. Slim loops, high susceptibility, high tunability, and reversible strain are the expected signature of an ergodic relaxor measured near its dielectric maximum; the manuscript itself attributes the x = 0.16 behavior to "its position within the relaxor response window near Tm." The burden, currently unmet, is to show what a distinct crossover explains that a conventional near-Tm optimum does not. The source data indicate that x = 0.16 lies very close to room-temperature Tm over common frequencies, which strengthens the near-Tm explanation; this should be reported explicitly and separated from the crossover claim.
The DFT cannot support the isotropy/flattened-landscape claim, which is the conceptual central claim. The argument rests on a single ordered 3 x 3 x 3 supercell per composition, with the cell fixed to XRD-derived lattice parameters. The computed net polarization magnitudes for x = 0.16 and x = 0.22 are essentially identical (48.17 vs 48.19 uC cm-2); the entire distinction is the orientation of one configuration's net dipole. Isotropy and energy-landscape flattening are properties of a statistical ensemble, not of a single ordered configuration: orientation is configuration-dependent, and a small ordered cell does not sample relaxor disorder, Sm-related charge-compensation chemistry, the A-site vacancies implied by the nominal formula, or nanoscale heterogeneity. The calculations should be demoted to qualitative, local-approximant illustrations of possible polarization tendencies, not presented as the foundation of the mechanism. If the isotropy claim is to be retained, it requires configurational averaging (e.g. multiple Ti/Sm arrangements or special-quasirandom/larger disordered cells).
x = 0.16 is not a unique optimum, and the trade-off claim needs statistics. It is not the maximum in strain (0.54% at x = 0.22, d33* ~2700 vs ~1550 pm V-1), and the manuscript's own text reports tunability "up to 80% for x >= 0.16," i.e. higher-Ti compositions match or exceed x = 0.16 (~77%); permittivity Tm rises monotonically with Ti. The defensible conclusion is a trade-off between sizeable response and reduced remanence/hysteresis under the selected conditions, not a uniquely flattened landscape. Because this is a quantitative trade-off argument, it should be supported by replicate pellets, uncertainty bars, and batch statistics; the present comparison rests on single specimens.
The local-structure evidence under-resolves the proposed polar texture. XRD is average-cubic for the Sm-doped series; Raman, PFM contrast, and HRTEM lattice images are consistent with increasing local polar coherence but do not by themselves establish a sequence from dynamic correlations to stabilized long-range domains. The authors own SAED/NBED show no superlattice spots, diffuse scattering, or spot splitting, and they concede the data “cannot directly support a definitive assignment of local PNR symmetry.” Given this, the persistent particle-like language (PNR density, field-driven PNR alignment, isolated local regions, PNR enlargement into long-range ferroelectric domains) and the statement that Raman/PFM show a “transition from isolated PNRs to long-range ferroelectric ordering” are too direct. The safer and more modern description is hierarchical, continuous, overlapping local polar correlations whose strength and reversibility change with Ti content.
Defect chemistry and processing are unaddressed confounds. The nominal Sm0.025Pb0.9625(Mg0.33Nb0.67)1-xTixO3 formula implies ~1.25% A-site vacancies. The added undoped controls are not defect-matched (Pb = 1, no vacancies), so they cannot disentangle Sm vs. vacancy vs. Ti contributions. PbO loss, sintered/relative density, porosity, and leakage current remain unquantified (only "dense" qualitatively); grain size (~5-10 um) and oxygen-vacancy-related internal bias are noted but are insufficient alone. These factors should be quantified or excluded before the trends are attributed mainly to Sm/Ti-driven polar-texture modulation.
At least one incomplete reference entry (e.g. Kavle et al., Adv. Mater. 36 (2024), missing its article/page number); check crystallographic/local-direction notation for consistency where it is used to support the structural interpretation; verify Nb stoichiometry notation throughout.
I cannot recommend acceptance in the present form. My recommendation is rejection in the present form, with encouragement to resubmit a substantially reframed manuscript or major revision only if the editors judge a path within this submission. The underlying study is sound and likely publishable, but its present identity depends on mechanistic and applied claims the data do not support. A defensible version would (i) reframe the work as a near-Tm composition-window trade-off study in one Sm/A-site-vacancy-modified PMN-PT series, (ii) demote the DFT to qualitative illustration or add configurational sampling, (iii) add replicates, error bars, absolute electrical metrics, and leakage separation, and (iv) replace

particle-like PNR language with hierarchical/continuous-correlation language.

Reviewer #3

(Remarks to the Author)
Although the authors have done their best to answer some of the comments, the overall quality of this manuscript is still not up to the standard for publication in this reputable journal. Polarization rotation is well known and the DFT calculation does not disclose any underlying mechanism to overcome the long-standing trade-off: compositions that deliver large electric-field-induced strain typically suffer from pronounced hysteresis. In fact, the current results show that the trade-off still exists: the x=0.22 sample shows larger strain than the x=0.16 sample, while it suffers from pronounced hysteresis.
1. The authors claimed that the best-balanced performance is achieved at x = 0.16. However, there is no indicator or figure of merit to show how the balanced performance is evaluated. Due to the lack of this evaluation, the readability of the manuscript quite poor, sometimes properties are reported for x = 0.16 and other times only the properties for x = 0.22 are demonstrated. There is a lack of comparison of the balanced performance.
2. Fig.3c, the current peaks appear in each quadrant of I-E loops at around 21 C may probably result from antiferroelectricity rather than ferroelectricity. More explanation is needed here.
3. The asymmetry in Smax under positive and negative fields was ascribed to internal bias fields caused by the oxygen vacancies. Supposing the oxygen vacancies should be randomly distributed, why is the Smax under positive field always larger than that under negative filed?
4. Supplementary Fig.3 shows the grain size varies significantly from sample to sample. The grain size of the samples should be measured and the effect of grain on d33 should not be neglected.

Version 2:

Reviewer comments:

Reviewer #2

(Remarks to the Author)
I thank the authors for the substantial revision. The manuscript has improved, and the authors have addressed many of the earlier concerns carefully. The composition-resolved dataset remains interesting. I would appreciate clarification of a few points important for reproducibility and proportionate interpretation.
The distinction between total, reversible, and irreversible tunability is central. The Methods section could define each quantity and state the AC amplitude\frequency, DC loading\unloading sequence, dwell time, field-removal procedure, and zero-bias recovery interval. Please clarify how the 78.2 +- 1.0% total and 76.4% reversible values at x = 0.16 relate and how the 64.0% reversible value at x = 0.22 was obtained. Uncertainties would be useful if replicate reversible data exist; otherwise, these values could be identified as representative values.
The DFT analysis needs clarification of the chemical configurations. The calculations use selected 3x3x3 periodic cells, but the Mg\Nb\Ti ordering model and the generation of the seven x = 0.16 configurations are unspecified; x = 0.08 and x = 0.22 remain represented by single configurations. Recent PMN–PT studies and the authors' observation that the broad spread among the seven x = 0.16 configurations itself demonstrates that the calculated polarization direction is sensitive to chemical configuration show that chemical disorder and residual short-range order influence local polar correlations. The cells also contain 3.70% Sm and no A-site vacancy rather than the nominal 2.5% Sm/A-site-vacancy chemistry. Thus, the calculations can illustrate possible polarization directions for selected local configurations but cannot establish statistical isotropy, dynamics, or a unique composition-driven sequence. Providing the initial and relaxed structures, briefly describing configuration construction, and retaining a qualitative local-approximant interpretation would strengthen the paper. Terms such as "comparatively isotropic", "dynamically reconfigurable" and DFT-guided identification or design may need qualification. Additional sampling is needed only if the stronger mechanistic claims are retained.
The Amm2 assignments for PMNT16, PMNT22, and PMNT16v are not documented in the Methods or the Supplementary refinement table. I would appreciate provision of the available model-comparison and refinement information or, alternatively, a more cautious structural description. The newly added current-density data lack details regarding stabilization time and leakage compensation. Please state the measurement protocol and clarify whether conduction affects the P–E loops and remanent-polarization values. "Minimal hysteresis" should likewise be quantified or softened.
Please also correct the swapped Fig. 2h and Fig. 2i citations, the unit in Fig. 3b from μC m−2 to μC cm−2, and "composition tunning" in the abstract. Unless saturation is demonstrated for each composition, Pmax may be more appropriate than Ps. Addressing these points would improve reproducibility and align the DFT and mechanistic interpretation with the evidence. I therefore recommend major revision.

Reviewer #3

(Remarks to the Author)
I'm satisfied with the revision.

Version 3:

Reviewer comments:

Reviewer #2

(Remarks to the Author)
I thank the authors for the careful revision. My previous substantive concerns have been addressed, and the interpretation is now appropriately aligned with the evidence. Only a small number of editorial corrections remain:
Please standardize the instrument model numbers: "Agilent 4294" and "Agilent 4284" should be given by their full designations, 4294A and 4284A, consistently in the Methods and Reporting Summary.
Please verify and correct the Raman instrument identification and excitation wavelength: "ViaTM confocal Raman microscope" appears to be an incomplete or malformed model name, and the stated 432 nm wavelength is likely a typographical error for 532 nm. Please make these details consistent in the Methods and Reporting Summary.
Please revise the malformed opening of the Fig. 3a caption, "Current* (Normalized current, I/Imax)-electric field", to a clear form such as "Normalized-current (I/Imax)-electric-field and P-E loops".
Reference 5 should give 2024, rather than 2023, as the year associated with Nat. Biotechnol. 42, 448-457. Reference 50 should include the complete author list: S. L. Dudarev, G. A. Botton, S. Y. Savrasov, J. Humphreys and A. P. Sutton.
The authors have addressed my earlier concerns; what remains is editorial and does not require further review.

Open Access This Peer Review File is licensed under a Creative Commons Attribution 4.0 International License, which permits use, sharing, adaptation, distribution and reproduction in any medium or format, as long as you give appropriate credit to the original author(s) and the source, provide a link to the Creative Commons license, and indicate if changes were made.
In cases where reviewers are anonymous, credit should be given to 'Anonymous Referee' and the source.
The images or other third party material in this Peer Review File are included in the article's Creative Commons license, unless indicated otherwise in a credit line to the material. If material is not included in the article's Creative Commons license and your intended use is not permitted by statutory regulation or exceeds the permitted use, you will need to obtain permission directly from the copyright holder.
To view a copy of this license, visit https://creativecommons.org/licenses/by/4.0/

Responses to Reviewers' Comments

We thank the reviewers for their helpful and constructive comments. Our responses to their specific points are provided below and all changes to the text are highlighted in the manuscript.

Reviewer #1:

In this work, the authors relatively large strain with low hysteresis in Sm-doped PMN-PT perovskite relaxor ferroelectric ceramics through polar nanoregion engineering. Specifically, the authors first calculated the impacts of Sm and Ti on the free energy landscape of PMN, then they designed a series of PMN-based ceramics with interesting electrostrictive and dielectric properties. Overall, I think this manuscript is worth for publication in Nature Communications as the authors addressed the following comments.

(1) For DFT calculations, the impact of Sm should be further discussed, e.g., its impact on polarization magnitude and orientation. Currently, only the concentration of Ti was emphasized.

Re: We thank the reviewer for this helpful suggestion. To further clarify the role of Sm, we have added DFT calculations for the undoped PMNT08 composition as a reference, because this composition shows a cubic average structure in the experimental XRD refinement and is therefore directly comparable with the Sm-doped x = 0.08 composition. PMNT16 and PMNT22 were not used for this comparison because they already show an orthorhombic Amm2 ferroelectric phase. The comparison between PMNT08 and SPMNT08 shows that Sm doping modifies both the magnitude and orientation of the calculated polarization. We have added relevant discussion in the revised manuscript and included the PMNT08 DFT results in Supplementary Fig. 1.

Revised context:

'To clarify the effect of Sm doping, an additional DFT calculation was performed for undoped PMNT08, which remains cubic experimentally (Supplementary Fig. 1). The calculated polarization of PMNT08 is dominated by a large P_z component, indicating a strong [001] polarization preference. After Sm substitution, this P_z dominance is reduced and the polarization components show redistribution, which suggests that Sm doping weakens polarization anisotropy and promotes a more multidirectional local polar configuration.

(2) The difference between calculation and experimental results is quite large for the PMN-based materials. It is understandable that the temperature and crystallographic orientations are different for calculations and experiments. However, how the authors use the calculation predicted data assists the design of real materials should be discussed.

Re: We thank the reviewer for this insightful comment regarding the large difference between DFT results and experimental data. In this study, the primary role of DFT

calculations was not to provide absolute numerical predictions, but to serve as a qualitative theoretical guide for understanding the polarization landscape's evolution. Specifically, DFT calculations identified that the composition x = 0.16 possesses a highly isotropic polarization distribution. This theoretical finding indicates a minimal energy barrier for polarization rotation, suggesting that this specific region could act as the crossover point for multifunctional properties. This prediction directly guided our experimental focus toward the ergodic–nonergodic transition regime. The experimental validation, where the x = 0.16 composition indeed exhibited the best-balanced composition within the investigated series of high strain and high tunability with low hysteresis.

Revised context:

This composition represents the point of energetic proximity among competing polarization orientations, lowering rotational barriers and enabling facile field-induced reorientation. Notably, while polarization magnitudes from DFT calculation are inherently higher due to 0 K ground-state conditions and idealized supercells, these results might serve as a strategic theoretical prediction. By mapping the orientation evolution of polarization, DFT suggests that x = 0.16 may have a more balanced local polarization tendency and minimized rotational barriers, enabling highly reconfigurable dipoles.

(3) The strain of PMN-0.22PT composition is quite large in polycrystalline ceramics. This point should be further discussed and highlighted in the abstract and conclusion.

Re: We thank the reviewer’s suggestion to highlight the high strain performance of the x = 0.22 composition. In the revised manuscript, we have added the discussion about physical origin of this high strain, which is the transition from dynamic PNRs to stabilized long-range ferroelectric ordering. In addition, we have now highlighted in the Abstract and Conclusion that the x = 0.22 composition achieves a giant strain of 0.54% at 2kV mm^{-1}.

Revised context:

In abstract

‘At the ergodic–nonergodic crossover (*x* = 0.16), the material exhibits ~0.31% reversible strain and ~77% dielectric tunability under 2 kV mm^{-1} with minimal hysteresis, arising from field-driven alignment of multidirectional PNRs without stabilization of long-range ferroelectric domains. Notably increasing the Ti content to *x* = 0.22 stabilizes long-range polar ordering and yields a high strain of 0.54% at 2 kV mm^{-1}.

In discussion

Slight asymmetry in S_{max} under positive and negative fields is attributed to internal bias fields caused by oxygen vacancies[20]. This high electromechanical response in *x* = 0.22 composition could be attributed to the field-induced enlargement and stabilization of ordered polar regions.

In conclusion

In contrast, higher Ti contents ($x > 0.16$) promote the enlargement of PNRs to long-range ferroelectric domains, resulting in increased remanent polarization and hysteresis losses. The composition with $x = 0.22$ achieves a high strain of 0.54% at 2 kV mm^{-1} (corresponding to d^*_{33} of 2700 pm V^{-1}). These findings demonstrate that controlled modulation of PNRs density and polarization anisotropy at ergodic and nonergodic boundaries enables the coexistence of dielectric agility and large electromechanical strain within a single perovskite platform.'

(4) The relationship between electrostrictive strain and dielectric tunnability isn't clear. The question here is that why the present work focuses on these two characteristics of PMN system.

Re: We thank the reviewer for this helpful comment. The electrostrictive strain and dielectric tunability are not necessarily governed by an identical microscopic mechanism in PMN-based systems. In particular, large electric-field-induced strain can involve field-induced domain growth, polarization switching, or phase-transition-related lattice distortion, whereas dielectric tunability is more directly associated with the field-dependent evolution of rotation of polarization, especially in compositions dominated by dynamic PNRs.

Our reason for focusing on both characteristics is that they represent two key functional responses of practical interest in the PMN system: electromechanical actuation and dielectric agility. By evaluating them together across the compositional crossover, we aim to clarify how the polarization state evolves from a PNR-dominated relaxor response toward a more domain-stabilized ferroelectric response, and to identify the composition that best balances large reversible strain, high tunability, and low hysteresis. This point has now been clarified in the revised Introduction.

Revised context:

'In PMN-based relaxor ferroelectrics, dielectric tunability and strain are both sensitive to the underlying polarization state. High dielectric tunability is typically associated with the response of PNRs, whereas large strain involves field-induced domain growth, polarization switching, or lattice distortion. Therefore, evaluating both responses together is important for understanding the relaxor ferroelectric crossover and for identifying compositions that best balance actuation capability, dielectric agility, and hysteresis. In this work, we implement a polarization landscape engineering strategy in Sm-doped PMN-PT ($Sm_{0.025}Pb_{0.9625}(Mg_{0.33}Nb_{0.67})_{1-x}Ti_xO_3$, SPMNT) by systematically modulating Ti content.

(5) The quality of TEM images isn't high. I don't think the marked regions are chemical ordered regions, since there isn't any superlattice diffraction point presented here. If they are not chemical ordered regions, the authors have to indicate what the "ordered" means.

Re: We agree with the reviewer that the absence of superlattice diffraction spots in the SAED patterns indicates that the marked regions are not chemical ordered regions. In fact, the term 'ordered' refers to lattice periodicity. In the revised manuscript, we have now clarified the terminology and replaced "ordered regions" with "regions showing locally ordered lattice patterns". Here, "ordered" refers only to the presence of relatively coherent lattice fringes or enhanced short-range lattice periodicity in the

HRTEM images, rather than chemical ordering. This interpretation is consistent with our Raman and PFM results, which collectively show the transition from isolated PNRs to long-range ferroelectric ordering with increasing Ti content.

Revised context:

'High resolution transmission electron microscopy (HRTEM) along the [111] crystallographic zone axis provides direct atomic scale insight into the evolution of local polar structure. In x = 0.08 composition, only a few isolated regions with locally ordered lattice patterns are observed, consistent with weak ferroelectric response and broad current peak observed in *I-E* loop. With increasing Ti content at x =0.16, the number of regions showing locally ordered lattice patterns increases. For the x = 0.22 composition, the locally ordered lattice patterns appear throughout most of the scanned area, with only small disorder regions present. Selected area electron diffraction (SAED) and nanobeam electron diffraction (NBED) were performed along the [211] zone axis, which show fundamental perovskite reflections, without clear superlattice spots, diffuse scattering, or spot splitting (Supplementary Fig. 10). Thus, the results cannot directly support a definitive assignment of local PNR symmetry, possibly due to the limited sensitivity of the technique. To further investigate the nanoscale structures, STEM-EDS mapping was performed for x = 0.16 (Supplementary Fig. 11). The HAADF-STEM images reveal nanoscale contrast fluctuations, indicating local structural heterogeneity within the matrix. Corresponding elemental maps show homogeneous distribution throughout the scanned area. These observations are consistent with the existence of local polar regions observed in HRTEM.'

Reviewer #2:

This manuscript reports a Sm-doped PMN-PT ceramic series and proposes that a composition near x = 0.16 offers a favorable balance of reversible strain, dielectric tunability, and low hysteresis. The topic is potentially interesting, and the search for a composition window that balances multiple functional metrics within one relaxor family is worthwhile.

In the comments below, I assess the manuscript in its present form and base my evaluation on the statements, quantitative assignments, and interpretations as they are currently written, rather than on probable intended meanings where these are not stated clearly.

One of the most important issues is the inconsistency in the strain / d33 assignment. The Abstract and Outlook assign ~0.31% strain at 2 kV mm−1 to x = 0.16, whereas the Results section assigns 0.54% to x = 0.16 and 0.31% to x = 0.22, with the corresponding d33* values shifting accordingly. As written, this creates ambiguity regarding which composition is actually being identified as the optimum. The manuscript would benefit from a careful consistency check across the Abstract, Results, figure discussion, captions, and Outlook.*

Re: We apologize for the mistakes. We have now conducted a thorough consistency check across the entire manuscript to ensure all values align with the experimental data shown in figures.

The study is framed as a Sm-doped PMN-PT design strategy, yet no undoped PMN-PT control series prepared and measured under comparable conditions is presented. As a result, it is difficult to distinguish cleanly among Ti-composition effects, Sm-substitution effects, and processing or microstructural contributions. Including such a control would significantly strengthen the central design argument.

Re: We agree with the reviewer's comment. To address this concern, we have now added control series of undoped $Pb(Mg_{0.33}Nb_{0.67})_{1-x}Ti_xO_3$ (x = 0.08, 0.16 and 0.22, corresponding to PMNT08, PMNT16, and PMNT22) with representative Ti contents in the Supporting Information. The additional data include XRD/Rietveld refinement results, temperature-dependent dielectric properties, and *P–E*/*I–E*/*S–E* measurements, allowing a direct comparison with the Sm-doped SPMNT compositions. These results are now presented in Supplementary Figs. S1, S2, S4, and S5.

Revised context:

'To further study the effect of Sm doping, undoped $Pb(Mg_{0.33}Nb_{0.67})_{1-x}Ti_xO_3$ compositions with *x* = 0.08, 0.16, and 0.22, denoted as PMNT08, PMNT16 and PMNT22, were also prepared and their refined XRD profiles are shown in Supplementary Fig. 2. PMNT08 can be refined using the cubic *Pm*-3*m* perovskite model, whereas PMNT16 and PMNT22 are better fitted using the orthorhombic *Amm*2 ferroelectric model. This comparison indicates that, in the undoped PMNT system, increasing Ti content promotes the development of a long-range ferroelectric phase, while the SPMNT compositions preserve an average cubic structure over the same compositional range. The result suggests that Sm doping helps destabilize long-range ferroelectric ordering and maintains a more disordered relaxor-like average structure.

For undoped PMNT system, PMNT08 shows a broad permittivity maximum with a T_m of *ca.* 20 °C and clear frequency dispersion, indicating relaxor-like behaviour. In contrast, PMNT16 and PMNT22 exhibit dielectric maximum at *ca.* 100 °C and 125 °C, respectively, with weaker frequency dispersion, consistent with the development of a more stable ferroelectric phase. This also in agreement with their room temperature *Amm*2 structural refinements and corresponding to the reported M_B phase in literature[29,30].

For undoped PMNT system, PMNT08 exhibits relatively high saturated polarization in *P-E* loop with current peaks near zero field in the I-E loop. In addition, compare to SPMNT16 and SPMNT22, PMNT16 and PMNT22 show much sharper switching current peaks in the *I-E* loops and typical ferroelectric *P-E* loops with obvious remanent polarization, indicating the stabilization of long-range ferroelectric domains.'

The current computational analysis does not appear sufficient to support the broader structural interpretation advanced in the manuscript. The calculations do not seem to represent the likely Sm-related charge-compensation chemistry, they use very small ordered supercells that do not meaningfully sample relaxor disorder, and they are performed with the lattice fixed to XRD-derived parameters despite the central importance of strain-polarization coupling in this system. Taken together, these limitations weaken strong inferences about polarization rotation, isotropy, or a flattened energy landscape. In its current form, the DFT section seems more suitable for a

limited discussion of local polarization tendencies based on small-cell models than for supporting the stronger PNR-scale and crossover claims made in the manuscript.

Re: We appreciate the reviewer's rigorous evaluation of our DFT analysis. Regarding polarization tendencies. We have therefore revised the manuscript to substantially tone down the DFT based interpretation. The calculations are now presented only as qualitative small-cell models that suggest local polarization tendencies with increasing Ti content. Stronger statements regarding PNR-scale disorder, polarization isotropy, flattened energy landscape, and the relaxor–ferroelectric crossover have been softened or reassigned to the experimental evidence. The use of XRD-derived parameters was a deliberate choice to investigate the electronic and polar response within the experimentally measured structural framework, ensuring that the calculated polarization rotation trends are relevant to our synthesized ceramics. In response to the reviewer's suggestion, we have modified the DFT section to emphasize its role as a tool for identifying local dipolar configurations and energy landscape flattening, which serves as a guiding framework for our PNR-scale interpretations. We have also stated the limitations of the model in the revised context.

Revised context:

'**2.1 First-Principles Insights into Local Polarization Tendencies**

To minimize artificial clustering effects, Ti substitutions were deliberately positioned at both edge and centre B-sites within the supercell (Fig. 1a-c). By utilizing XRD-derived lattice parameters as constraints, the simulations focus on resolving how Ti-content modulates the local polarization anisotropy within the experimental structural framework.

The resulting polarization vectors (P_x, P_y and P_z) and their unit vectors (μ_x, μ_y and μ_z) map a clear evolution of the local dipolar behaviour (Fig. 1f). At x = 0.08, the polarization vector lies predominantly in the x-y plane, … This composition represents the point of energetic proximity among competing polarization orientations, lowering rotational barriers and enabling facile field-induced reorientation. To clarify the effect of Sm doping, an additional DFT calculation was performed for undoped PMNT08, which remains cubic experimentally (Supplementary Fig. 1). The calculated polarization of PMNT08 is dominated by a large P_z component, indicating a strong [001] polarization preference. After Sm substitution, this P_z dominance is reduced and polarization components show redistribution, which suggests that Sm doping weakens polarization anisotropy and promotes a more multidirectional local polar configuration. Notably, while polarization magnitudes from DFT calculation are inherently higher due to 0 K ground-state conditions and idealized supercells, these results serve as a strategic theoretical compass. The DFT results suggest that the x = 0.16 composition may possess a more balanced local polarization tendency, enabling highly reconfigurable dipoles. It should be noted that these 3 × 3 × 3 ordered supercell calculations are simplified models and do not fully capture the chemical disorder, charge-compensation chemistry, or nanoscale heterogeneity inherent to PMN-based relaxor ferroelectrics. Therefore, the present DFT results should be interpreted as qualitative indicators of local polarization tendencies with increasing Ti content, rather than as a direct atomistic representation of PNR-scale disorder or the relaxor ferroelectric crossover.'

Language around PNRs should also be made more cautious. The manuscript repeatedly uses wording suggesting that discrete, isolable PNR objects and their density or alignment are being engineered directly. This interpretation seems stronger than the present methods can firmly establish, and it does not engage fully with more recent PMN / PMN-PT literature, which often favors hierarchical polar correlations with smooth spatial variation or continuous and overlapping polarization textures over a simple independent-PNR picture. The argument would be more convincing if it more clearly separated what is directly measured from what is inferred.

Re: We agree with the reviewer that the traditional 'discrete PNR' model is an idealized simplification and that the actual polar structure likely involves hierarchical correlations and continuous spatial variations in PMN-PT system according to updated literature [*Adv. Funct. Mater.*, 30, 2006823, (2020); Nature, 546, 391–395 (2017)]. In the revised manuscript, we have adopted more cautious language to describe these features. We have shifted the emphasis from 'engineering discrete PNRs' to 'modulating the polarization landscape' and 'polarization textures'. We have clarified that 'PNRs' are used as a conceptual framework to interpret the observed enhancement in polar fluctuations and spatial disorder. We have separated our direct experimental observations from the mechanistic inferences regarding dipolar reconfigurability. This adjustment ensures our discussion remains physically rigorous while maintaining the clarity of our design strategy based on polarization isotropy and energetic proximity.

Revised context:

‘For low Ti compositions ($x \leq 0.12$), the polarization-electric field (*P-E*) loops are narrow and the current peaks in current-electric field (*I-E*) loops are weak (Fig. 3a), indicating polarization response dominated by highly dynamic and localized polar correlations, characteristic of an ergodic relaxor state. As the Ti content increases to $x = 0.16$, the nonlinear *P-E* hysteresis loops become more pronounced with higher polarization response, suggesting enhanced magnitude of polarization and increased configurational diversity within the polarization landscape….. The best-balanced performance of $x = 0.16$ is attributed to the combined effect of its position within the relaxor response window near T_m and the composition-driven evolution of local polar behaviour. In this composition, local polar regions are sufficiently developed to give strong dielectric and strain responses but remain reversible without strong long-range ferroelectric stabilization. With further Ti substitution, as in $x = 0.22$, the polar state becomes more stabilized, producing larger strain but also higher remanent polarization and more pronounced switching features.

For the $x = 0.16$ composition, the PFM phase image shows a much stronger contrast with irregular nanoscale patterns distributed across the surface. The phase signal exhibits large fluctuations, and the amplitude response becomes more pronounced along the line profile, indicating enhanced local polar response and a high density of dynamic polar regions. For $x = 0.22$ composition, … Moreover, while both compositions show polar features, the $x = 0.16$ composition reveals disrupted and localized polar correlations, suggesting localized polar correlations that have not yet developed into continuous ferroelectric domains. The contrast with the more continuous and interconnected polar network in $x = 0.22$, indicating enhanced

structural coherence and extended polar correlations, consistent with a transition toward a stabilized ferroelectric-like state.[30,33]'

The structural interpretation also appears more specific than the data strictly support. All compositions are refined using an average cubic Pm-3m model, yet the manuscript later makes more specific claims regarding local phase competition, increasing ferroelectric order, and domain stabilization. Raman, PFM, and HRTEM may indeed support increasing local polar coherence with Ti content, but they do not seem sufficient, on their own, to establish the full structural specificity of the current narrative. It would therefore be helpful either to soften these interpretations or to provide additional direct symmetry-sensitive evidence.

Re: The authors appreciate the reviewer's critical point regarding the structural interpretation. Our XRD refinements yielded an average cubic model due to the limited correlation length of the polar distortions in these relaxor systems. However, the claims regarding 'local phase competition' and 'domain stabilization' are not based solely on average diffraction, but on the convergent evidence from local-structure-sensitive techniques including Raman, dielectric and ferroelectric test. In the revised manuscript, we have carefully softened our terminology to distinguish between the 'average cubic symmetry' and 'local polar distortions'.

Revised context:

'The emergence of this feature indicates onset of enhanced local polar correlations, where the energy barriers between different polar orientations begin to increase. This suggests a gradual transition from an ergodic state toward localized symmetry-breaking behaviour. Although the average structure remains cubic, these features indicate the initial stabilization of the extended polar regions.[30,33] With increasing Ti concentration, the collinear state, corresponding to the temperature region between the anomaly and the main permittivity peak, extends over a broader temperature range, supported by the temperature-dependent dielectric permittivity in Fig. 2i.

Accordingly, the evolution of the Raman spectra demonstrates a crossover from relaxor-like behaviour dominated by disorder octahedral breathing to a more coherent polar framework, as evidenced by hardened C-modes and a strengthened 740 cm^{-1} peak, which is indicative of an increased local polar coherence and the emergence of long-range polar order.

Despite the average cubic symmetry revealed by XRD, composition with $x < 0.16$ exhibit dynamic polar nanoregions, whose local polar coherence and orientational preference enhanced with Ti substitution, enabling rapid and reversible dipolar reorientation under electric bias.'

The electrical evidence would benefit from stronger validation. The key P-E, I-E, and S-E loops are mainly presented at 1 Hz, without a clearly documented study of frequency dependence, leakage contributions, cycling stability, or reproducibility. Because these data are used both to motivate the mechanism and to support application-facing performance claims, a more thorough electrical characterization would strengthen the paper substantially. Relatedly, the pellets are described only as approximately 1-2 mm thick before sintering, while the main figures of merit are expressed in kV mm-1 and d33 = Smax/Emax. Exact final sample thicknesses and*

field uncertainty are not clearly documented for each measured specimen. Since the central comparison depends directly on electric field, clearer reporting here would be important.

Re: We fully agree with the reviewer for the constructive suggestions. To address the concerns, we have performed additional characterizations on the x = 0.16 composition, now included as Supplementary Fig. S7 to study the frequency dependence *I-E*, *P-E* and *S-E* loops and the cycling stability. The low current level and stable P–E response indicate that leakage contributions are limited under the measurement conditions. We have added the relevant discussion in the manuscript accordingly. We have also added the details of sample preparation in the revised Experimental Section.

Revised context:

'The *P-E* and *S-E* loops remain nearly unchanged from 0.5 Hz to 10 Hz, indicating that the polarization switching and electromechanical strain are dominated by fast PNR reorientation (Supplementary Fig. S7). Furthermore, the *x* = 0.16 composition maintains a large strain of 0.29% after 100 cycles. The minor observed decrease is likely attributed to localized pinning of polar correlations by internal bias fields or defect dipoles.

Finally, the pellets were sintered at 1200 °C for 2 hours. Parallel syntheses were carried out and confirmed the reproducibility of the results.

For electrical measurements, sintered pellets were ground to rectangular plates of approximate dimensions 4 × 4 mm^2 and thickness of 0.30 ± 0.01 mm and silver paste (Gwent Electronic Materials Ltd. Pontypool, UK) was applied to the parallel faces of ceramic pellets, which were then heated to 450 °C for 10 mins to form the electrodes.'

The proposed optimum at x = 0.16 is also not yet established robustly. The paper treats this composition as a special optimum, but that conclusion is not strongly supported statistically. There are no replicate pellets, no uncertainty bars, no batch-to-batch variation, no finer composition spacing near the proposed optimum, and no strain data shown for the compositions x = 0.14 and x = 0.18. At present, the optimum appears suggestive rather than firmly established. More broadly, the manuscript argues for a special crossover composition near x = 0.16, but it does not yet convincingly show why the observed balance of tunability and strain could not be explained largely as a near-Tm effect within the relaxor response window. In its current form, the paper does not clearly separate a distinct crossover mechanism from a more conventional near-Tm optimization scenario.

Re: We thank the reviewer's comments. To address these concerns, we have now provided the field-induced strain data for the intermediate compositions x = 0.14 and 0.18 (Fig. 3). We acknowledge that the present study does not include statistical uncertainty analysis, or finer composition intervals around x = 0.16. The focus here is to show that x = 0.16 composition is considered the optimum within the investigated series for balancing large reversible strain, high dielectric tunability, low remanent polarization, and low hysteresis. Nevertheless, we have revised to describe x = 0.16 as the "best balanced composition within the investigated series", rather than a statistically definitive optimum.

We agree with the reviewer, and we have now explained the mechanism from a more conventional near –Tm optimization scenario. Specifically, we now clarify that the near-*Tm* response and the proposed crossover behaviour are closely related: Ti substitution gradually drives the system from a highly dynamic relaxor state toward a more stabilized polar state, and x = 0.16 lies in the composition range where the field-induced dielectric, polarization, and strain responses are strongly enhanced while excessive remanent polarization and hysteresis remain limited. We have modified the manuscript accordingly.

Revised context:

'Finally, the pellets were sintered at 1200 °C for 2 hours. Parallel syntheses were carried out and confirmed the reproducibility of the results.

The best-balanced performance of $x = 0.16$ is attributed to the combined effect of its position within the relaxor response window near T_m and the composition-driven evolution of local polar behaviour. In this composition, local polar regions are sufficiently developed to give strong dielectric and strain responses but remain reversible without strong long-range ferroelectric stabilization. With further Ti substitution, as in $x = 0.22$, the polar state becomes more stabilized, producing larger strain but also higher remanent polarization and more pronounced switching features.'

There are also several secondary issues that should be corrected carefully. The nominal chemistry is written inconsistently (Nb0.67 versus Nb0.66) across the manuscript and supplement, which is important in a composition-sensitive study. The bibliography appears to contain duplicate entries and at least one incomplete-looking reference('Li et al.' appears as Refs 17 and 29; 'Huangfu et al.' as Refs 20 and 36; the authors own 2025 high-strain paper appears as Refs 30 and 32.). The ionic-radius argument in the unit-cell-volume discussion may need to be revisited, both in terms of coordination-number choice and numerical consistency. Topology images of PFM should be corrected to topography images, and the crystallographic direction notation should be made consistent. The exact tunability protocol details, current normalization, and strain-sensing methodology should also be reported more explicitly.

Re: We are grateful for the reviewer's careful check of the details. We have modified the stoichiometry to $Nb_{0.67}$ throughout the manuscript. Duplicate references (Refs 17/29, 20/36, and 30/32) have been merged, and the completeness of all entries has been verified. We have corrected the coordination number (CN) from 8 to 6 for the B-site cations in our discussion. 'Topology' has been corrected to 'Topography' in the PFM section. We have also added more details regarding the tunability, the method of current normalization, and the strain-sensing instrumentation in the revised experimental section.

Reviewer #3:

The authors developed a polarization landscape engineering strategy to control the anisotropy of PNRs in Sm-doped PMN-PT solid solution by tuning Ti concentration. They achieved 0.31% reversible strain and 77% dielectric tunability under an electric

field of 2 kV/mm with minimal hysteresis. The novelty of the current work is not sufficient to warrant the publication in this journal. Besides, the experimental work is not sufficient to support the conclusion.

1. Two lines above Equation (1), the authors claim that the A-site cations and O anions are the main contributors to the spontaneous polarization. Is this conclusion valid for all the different Ti concentrations studied? What is the underlying mechanism that the varied concentration of Ti will affect the polarization direction or the displacement direction of A-site cations? Intuitively, I would expect that as Ti increases in concentration, the off-center displacement direction of Ti cations will be affected.

Re: We thank the reviewer for this important comment. The conclusion that A-site cations and O anions make the larger contribution to the calculated spontaneous polarization is only based on the representative DFT models studied here with different Ti concentration of x = 0.08, 0.16, and 0.22. We also agree that Ti off centering is important. In the revised manuscript, we now state that the spontaneous polarization arises from coupled displacements of the A-site, B-site, and oxygen sublattices. Increasing Ti content modifies B-O covalency, off-centering, and BO_6 octahedral distortion, which in turn changes the coupling between B-site displacement and A-site/O-sublattice shifts. We have revised the text to clarify this point.

Revise context:

'For the representative DFT models examined compositions with variation of Ti concentration, the calculated polarization arises from coupled displacements of the A-site, B-site and oxygen sublattices. Although the Pb/Sm and oxygen sublattices make substantial contributions, B-site off-centering and BO_6 octahedral distortion also play an important role in tuning the polarization direction.'

2. As Ti concentration increases (x>0.16), the authors claim the existence of local orthorhombic and tetragonal phases, which facilitates the easier polarization rotations within PNRs. Is there any experimental evidence for the local orthorhombic and tetragonal phases? Are those local polar phases due to composition inhomogeneities or fluctuations?

Re: We thank the reviewer for this important comment. We agree that the present data do not directly prove the coexistence of well-defined local orthorhombic and tetragonal phases, although the M_B, M_C (orthorhombic) and tetragonal phases are reported in literature on PMN-xPT phase diagram [*Adv. Funct. Mater.,* 30, 2006823, (2020)*; PHYSICAL REVIEW B*, **74**, 024101, (2006)]. We have therefore revised the wording to avoid overstatement based on our data. These distortions are inferred from the dielectric anomaly and the evolution of local polar responses, while the average structure remains cubic according to XRD. They are more likely associated with nanoscale polar structures.

Revised context:

'The emergence of this feature indicates the onset of enhanced local polar correlations, where the energy barriers between different polar orientations begin to increase. This suggests a gradual transition from an ergodic state toward localized symmetry-

breaking behaviour. Although the average structure remains cubic, these features indicate the initial stabilization of the extended polar regions.[30,33]'

3. What is the local symmetry of the PNRs observed in Fig.4? Detailed microstructural characterizations on those PNRs should be conducted, which include diffraction by nano-beam. HAADF-STEM should be conducted to extract the information about the atomic displacement on the region observed.

Re: The reviewer asked a very important question, the nature of PNRs in relaxor ferroelectrics. According to updated literature [*Adv. Funct. Mater.*, 30, 2006823, (2020); *Nature*, 546, 391–395 (2017)], In $Pb(Mg_{1/3}Nb_{2/3})O_3$–$PbTiO_3$ relaxor ferroelectrics, polar nanoregions are now understood as highly correlated nanoscale multidomain structures rather than isolated polar clusters embedded in a non-polar matrix. These ultrafine polar domains (~2–10 nm) exhibit dynamic polarization fluctuations resembling a "polar slush" state. Their formation originates from local chemical and structural heterogeneity, including chemical disorder, oxygen octahedral tilting/distortion, lattice strain disorder, and polarization fluctuations. These heterogeneities correlate with low-angle nanodomain walls and monoclinic-like local distortions, which facilitate polarization rotation and domain-wall motion, collectively leading to the relaxor state and giant piezoelectric response in PMN–PT.

The exact nature of polar nanoregions in relaxor ferroelectrics remains under active research and is still evolving. Although substantial progress has been made, particularly regarding nanoscale multidomain structures, local chemical–structural heterogeneity, and dynamic polarization correlations, a complete and universally accepted microscopic description of PNRs has not yet been established.

The present study does not aim to resolve the full microscopic nature of PNRs, but rather to correlate evolving local polar correlations with macroscopic functional responses.

Nevertheless, we have now carried out selected area electron diffraction (SAED), nanobeam electron diffraction (NBED) and HAADF-STEM work, shown in Fig. S10 and S11, according to the reviewer's valuable comments. The relevant discussion has been added in the revised manuscript.

Revised context:

'Selected area electron diffraction (SAED) and nanobeam electron diffraction (NBED) were performed along the [211] zone axis, which show fundamental perovskite reflections, without clear superlattice spots, diffuse scattering, or spot splitting (Supplementary Fig. 10). Thus, the results cannot directly support a definitive assignment of local PNR symmetry, possibly due to the limited sensitivity of the technique. To further investigate the nanoscale structures, STEM-EDS mapping was performed for x = 0.16 (Supplementary Fig. 11). The HAADF-STEM images reveal nanoscale contrast fluctuations, indicating local structural heterogeneity within the matrix. Corresponding elemental maps show homogeneous distribution throughout the scanned area. These observations are consistent with the existence of local polar regions observed in HRTEM.'

4. The authors emphasize the minimal hysteresis achieved in x=0.16. However, why

do they only report the temperature dependance of the P-E hysteresis loop of the sample at x=0.22 (Fig.3)?

Re: We thank the reviewer for this helpful comment. The temperature dependence of *P–E*/*I–E* loops were measured for the x = 0.22 composition because this composition exhibits the most distinct and whole informative switching evolution with temperature. As shown in Fig. 3c and Supplementary Fig. S6, x = 0.22 shows clear ferroelectric-like hysteresis loops with two sharp switching current peaks at low temperatures. Upon heating, additional current peaks appear and the loops become progressively narrower, indicating a transition from conventional ferroelectric switching to multi-step field-induced polar ordering. At higher temperatures, the current peaks broaden and weaken, and the *P–E* loops become nearly linear, consistent with a gradual transition toward relaxor-like and eventually paraelectric behaviour. We have revised the text to clarify this information.

Revised context:

'The temperature dependence of *I-E*/*P-E* loops of *x* = 0.22 composition were studied because this composition exhibits clear and whole switching-current features, making it suitable for tracking the evolution of polarization switching with temperature. These measurements reveal a progressive transition from a ferroelectric state at low temperature to relaxor-ferroelectric behaviour at higher temperature'

Responses to Reviewers' Comments

We thank the reviewers for their helpful and constructive comments. Our responses to their specific points are provided below and all changes to the text are highlighted in the manuscript.

Reviewer #2:

I thank the authors for the careful revision and detailed response. The manuscript is clearer, and several additions are genuinely useful: the selected undoped PMN-PT controls, the more cautious DFT framing, the moderated PNR language, the added frequency\cycling data, and the intermediate-composition strain data. I also appreciate that x = 0.16 is now described more carefully as a best-balanced composition within the investigated series, with its proximity to the relaxor response window acknowledged more explicitly.

I want to be clear about what I think is strong here, because it matters for the recommendation. The core functional result, simultaneously achieving sizeable reversible electrostrain, high dielectric tunability, and low remanence/hysteresis in a single bulk ceramic, is a worthwhile and non-trivial achievement, and the composition-resolved dataset is a real contribution.

My concern is not the data, but the interpretive framing built on top of it. As written, the paper is presented as a mechanism-resolved demonstration of "PNR engineering" producing an ergodic-nonergodic crossover with a uniquely flattened polarization landscape, and as a route to low-loss multifunctional device performance. The evidence more securely supports a narrower and still valuable message: near-Tm composition-window optimization of strain, dielectric tunability, and low remanence in one Sm/A-site-vacancy-modified PMN-PT ceramic series. The title, abstract and conclusions should be reframed accordingly and should avoid implying a fully mechanism-resolved PNR-engineering strategy, a firmly established ergodic-nonergodic crossover, or low-loss multifunctional device performance.

Re: We thank the reviewer for the constructive and balanced assessment of the revised manuscript. The title has been revised to "Large strain and high reversible dielectric tunability in Sm-modified PMN-PT ceramics" to emphasize the functional performance. The abstract and conclusion have also been revised to reduce overstatement and to present the DFT results as qualitative indicators of local polarization tendencies. We have softened the mechanistic language by replacing or qualifying terms such as "PNR engineering," "uniquely flattened polarization landscape," and "long-range ferroelectric domain formation." The structural characterization is now discussed more cautiously in terms of evolving local polar correlations, spatial coherence, and polarization tendency, consistent with recent descriptions of relaxor ferroelectrics as hierarchical and continuous polar correlations [Nature 546, 391–395 (2017). *Nat. Commun.* **10**, 2728 (2019)]. We have further clarified the composition-dependent functional trends. The revised manuscript now highlights that the *x* = 0.16 composition provides the highest reversible dielectric tunability of 76.4% together with 0.31% strain and minimal hysteresis under 2 kV mm^{-1}, whereas *x* = 0.22 achieves a larger strain of 0.54% but shows increased hysteresis

loss and a reduced reversible tunability of 64.0%. This comparison supports the revised interpretation that $x = 0.16$ represents an optimized composition window where local polar correlations are sufficiently developed to provide strong dielectric and strain responses, while remaining largely reversible without strong remanent polar stabilization.

The proposed mechanism is not yet separated from conventional near-Tm relaxor behavior. Slim loops, high susceptibility, high tunability, and reversible strain are the expected signature of an ergodic relaxor measured near its dielectric maximum; the manuscript itself attributes the x = 0.16 behavior to "its position within the relaxor response window near Tm." The burden, currently unmet, is to show what a distinct crossover explains that a conventional near-Tm optimum does not. The source data indicate that x = 0.16 lies very close to room-temperature Tm over common frequencies, which strengthens the near-Tm explanation; this should be reported explicitly and separated from the crossover claim.

Re: We thank the reviewer for this important comment. The near room-temperature T_m of $x = 0.16$ contributes to high dielectric response, slim P–E loop, high reversible tunability and reversible strain. In addition, we have clarified that the crossover discussion is used to describe the Ti-dependent evolution of polar-state stability across the series. The near-T_m condition does not fully account for the Ti-dependent emergence of the lower-temperature dielectric anomaly and four current peak switching, or for the temperature-dependent evolution observed in $x = 0.22$ compositions. These features indicate a change in polar-state stability across the series. In x = 0.22, the additional current peaks in *I-E* loops appear around the lower-temperature dielectric anomaly and gradually evolve with temperature before merging into broad features near T_m. This behaviour is consistent with an ergodic/nonergodic polar-state crossover involving reversible field-induced ordering of polar regions. Similar field-induced relaxor-to-ferroelectric transitions have been reported in PMN-PT and related relaxor systems [*Nat. Commun.*, **14**, 2414, (2023), *J. Mater. Chem. C*, **9**, 3204-3214 (2021), *J. Appl. Phys.*, **110**, 074109 (2011)]. We have revised the manuscript on page 10, accordingly.

The DFT cannot support the isotropy/flattened-landscape claim, which is the conceptual central claim. The argument rests on a single ordered 3 x 3 x 3 supercell per composition, with the cell fixed to XRD-derived lattice parameters. The computed net polarization magnitudes for x = 0.16 and x = 0.22 are essentially identical (48.17 vs 48.19 uC cm-2); the entire distinction is the orientation of one configuration's net dipole. Isotropy and energy-landscape flattening are properties of a statistical ensemble, not of a single ordered configuration: orientation is configuration-dependent, and a small ordered cell does not sample relaxor disorder, Sm-related charge-compensation chemistry, the A-site vacancies implied by the nominal formula, or nanoscale heterogeneity. The calculations should be demoted to qualitative, local-approximant illustrations of possible polarization tendencies, not presented as the foundation of the mechanism. If the isotropy claim is to be retained, it requires configurational averaging (e.g. multiple Ti/Sm arrangements or special-quasirandom/larger disordered cells).

Re: We agree with the reviewer that the DFT results cannot, by themselves, establish statistical isotropy or a flattened polarization landscape. Therefore, in the revised manuscript, we have demoted the DFT discussion and now present the calculations as qualitative local-approximant illustrations of possible polarization tendencies from simplified 3 × 3 × 3 ordered supercells with fixed XRD-derived lattice parameters. We have also softened the language from “flattened landscape” to “local polarization tendency” and “composition-dependent polar-state evolution.” Moreover, in order to reduce reliance on a single ($x = 0.16$) configuration, seven additional randomly generated configurations were relaxed, showing a distribution of intermediate polarization orientations (Supplementary Fig. 1). We now describe this as a comparatively broad local polarization tendency. We have now revised the manuscript on page 5 accordingly.

x = 0.16 is not a unique optimum, and the trade-off claim needs statistics. It is not the maximum in strain (0.54% at x = 0.22, d33 ~2700 vs ~1550 pm V-1), and the manuscript's own text reports tunability "up to 80% for x >= 0.16," i.e. higher-Ti compositions match or exceed x = 0.16 (~77%); permittivity Tm rises monotonically with Ti. The defensible conclusion is a trade-off between sizeable response and reduced remanence/hysteresis under the selected conditions, not a uniquely flattened landscape. Because this is a quantitative trade-off argument, it should be supported by replicate pellets, uncertainty bars, and batch statistics; the present comparison rests on single specimens.*

Re: We thank the reviewer for this helpful comment. To better support the trade-off discussion, we have revised Fig. 3g to distinguish the reversible and irreversible components of dielectric tunability, which shows that the $x = 0.16$ composition exhibits the highest reversible tunability while maintaining high strain and low remanent polarization. Therefore, $x = 0.16$ is identified as the best-balanced composition for reversible multifunctional response. To assess specimen-to-specimen reproducibility, the tunability of six independently prepared $x = 0.16$ specimens from different batches was measured at 2 kV mm^{-1}. The mean tunability was 78.2 ± 1.0 %, with a relative standard deviation of 1.3%, confirming good reproducibility of the reported response. The corresponding discussion has been revised on page 9-10.

The local-structure evidence under-resolves the proposed polar texture. XRD is average-cubic for the Sm-doped series; Raman, PFM contrast, and HRTEM lattice images are consistent with increasing local polar coherence but do not by themselves establish a sequence from dynamic correlations to stabilized long-range domains. The authors own SAED/NBED show no superlattice spots, diffuse scattering, or spot splitting, and they concede the data “cannot directly support a definitive assignment of local PNR symmetry.” Given this, the persistent particle-like language (PNR density, field-driven PNR alignment, isolated local regions, PNR enlargement into long-range ferroelectric domains) and the statement that Raman/PFM show a “transition from isolated PNRs to long-range ferroelectric ordering” are too direct. The safer and more modern description is hierarchical, continuous, overlapping local polar correlations whose strength and reversibility change with Ti content.

Re: We agree with the reviewer. In the revised text, Raman, PFM and HRTEM are described as evidence for a gradual increase in the strength, spatial coherence and stability of local polar correlations with increasing Ti content. The discussion is now framed in terms of hierarchical and continuous local polar correlations whose field

responsiveness and reversibility change with Ti content, consistent with recent descriptions of relaxor ferroelectrics as complex polar-correlation networks [Nature 546, 391–395 (2017); *Nat. Commun.* **10**, 2728 (2019)].

Defect chemistry and processing are unaddressed confounds. The nominal Sm0.025Pb0.9625(Mg0.33Nb0.67)1-xTixO3 formula implies ~1.25% A-site vacancies. The added undoped controls are not defect-matched (Pb = 1, no vacancies), so they cannot disentangle Sm vs. vacancy vs. Ti contributions. PbO loss, sintered/relative density, porosity, and leakage current remain unquantified (only "dense" qualitatively); grain size (~5-10 um) and oxygen-vacancy-related internal bias are noted but are insufficient alone. These factors should be quantified or excluded before the trends are attributed mainly to Sm/Ti-driven polar-texture modulation.

Re: We agree with the reviewer on this point. In the revised version, we have added quantitative relative density, grain-size and leakage-current data. All SPMNT ceramics show relative densities above 95%, average grain sizes of approximately 3.9-9.1 μm, and current densities of 20-50 μA cm^{-2} under 2 kV mm^{-1}, indicating that the observed trends are not dominated by porosity or severe leakage. We have also added an A-site-deficient control composition, $Pb_{0.9875}(Mg_{1/3}Nb_{2/3})_{0.84}Ti_{0.16}O_3$ (PMNT16v) to examine the vacancy effect. PMNT16v shows a lower T_m and stronger frequency dispersion than PMNT16, suggesting that A-site vacancies promote a more relaxor-like response. However, PMNT16v still exhibits an Amm2 ferroelectric average structure, whereas SPMNT16, which contains the same nominal A-site vacancy concentration but also includes Sm substitution, shows an average cubic structure. This comparison indicates that Sm substitution also plays an important role in destabilizing long-range ferroelectric ordering. The *P*–*E* loops do not show a pronounced coercive field shift. Thus *S*–*E* asymmetry is more likely associated with a weak electromechanical bias arising from asymmetric field induced polar reorientation, residual stress, or oxygen vacancy related defect structures. The manuscript has been revised on page 7-11, accordingly.

At least one incomplete reference entry (e.g. Kavle et al., Adv. Mater. 36 (2024), missing its article/page number); check crystallographic/local-direction notation for consistency where it is used to support the structural interpretation; verify Nb stoichiometry notation throughout.

Re: We thank the reviewer for these careful comments. We have checked and corrected the reference list. We have also revised the crystallographic/local-direction notation where the polarization tendencies are described using ⟨110⟩ and ⟨001⟩. Finally, we have carefully checked the Nb stoichiometry notation throughout the manuscript and Supplementary Information. The PMN composition is consistently written as $PbMg_{1/3}Nb_{2/3}O_3$.

I cannot recommend acceptance in the present form. My recommendation is rejection in the present form, with encouragement to resubmit a substantially reframed manuscript or major revision only if the editors judge a path within this submission. The underlying study is sound and likely publishable, but its present identity depends on mechanistic and applied claims the data do not support. A defensible version would (i) reframe the work as a near-Tm composition-window trade-off study in one Sm/A-site-vacancy-modified PMN-PT series, (ii) demote the DFT to qualitative illustration or

add configurational sampling, (iii) add replicates, error bars, absolute electrical metrics, and leakage separation, and (iv) replace particle-like PNR language with hierarchical/continuous-correlation language.

Re: We thank the reviewer for this constructive assessment. In the revised manuscript, we have reframed the work from a mechanism-led "PNR engineering" study to a composition-window study in Sm-modified PMN-PT ceramics. We have added further experimental evidence, including the PMNT16v comparison, reversible/irreversible tunability analysis, leakage-current information and reproducibility assessment for x = 0.16 composition. We have also added seven additional randomly generated (x = 0.16) configurations for DFT calculations. The DFT results are now presented as qualitative local-approximant illustrations. In addition, particle-like PNR language has been replaced with more cautious descriptions of hierarchical, continuous local polar correlations and polar-state evolution, consistent with recent descriptions of relaxor ferroelectrics [Nature 546, 391–395 (2017); *Nat. Commun.* **10**, 2728 (2019)].

Reviewer #3:

Although the authors have done their best to answer some of the comments, the overall quality of this manuscript is still not up to the standard for publication in this reputable journal. Polarization rotation is well known and the DFT calculation does not disclose any underlying mechanism to overcome the long-standing trade-off: compositions that deliver large electric-field-induced strain typically suffer from pronounced hysteresis. In fact, the current results show that the trade-off still exists: the x=0.22 sample shows larger strain than the x=0.16 sample, while it suffers from pronounced hysteresis.

1. The authors claimed that the best-balanced performance is achieved at x = 0.16. However, there is no indicator or figure of merit to show how the balanced performance is evaluated. Due to the lack of this evaluation, the readability of the manuscript quite poor, sometimes properties are reported for x = 0.16 and other times only the properties for x = 0.22 are demonstrated. There is a lack of comparison of the balanced performance.

Re: We thank the reviewer for this helpful comment. In the revised manuscript, we have revised Fig. 3g to separate the reversible and irreversible components of dielectric tunability and added a clearer comparison with strain and remanent polarization. The revised analysis shows that (x = 0.16) exhibits the highest reversible tunability while maintaining sizeable electrostrain and low remanence/hysteresis, whereas (x = 0.22) gives larger strain but increased remanent polarization and a larger irreversible tunability component. The corresponding discussion has been revised to make the composition comparison and selection criteria clearer.

2. Fig.3c, the current peaks appear in each quadrant of I-E loops at around 21 C may probably result from antiferroelectricity rather than ferroelectricity. More explanation is needed here.

Re: We thank the reviewer for this comment. We do not assign this behaviour to true antiferroelectricity which normally requires antipolar structural ordering and is often associated with a low permittivity response [*J Am Ceram Soc.* **104** 3775–3810 (2021)], In addition, the present structural and local structure results do not provide evidence for antipolar ordering. Instead, in this PMN-PT-based relaxor system, the four current peaks are more interpreted as arising from a reversible field-induced transition between a weakly ordered relaxor state and a field-aligned polar state. During field loading, weakly ordered relaxor state are driven into a more ordered polar configuration, while during field unloading the induced polar state relaxes back toward the initial relaxor state, which is also consistent with evidence in papers on relaxor PMN ferroelectrics [*Journal of Materiomics* **7**, 1143-1152 (2021); *J. Am. Chem. Soc.*, **147**, 42909−42917 (2025)]. We have revised the manuscript to make this clear on page 8.

3. The asymmetry in Smax under positive and negative fields was ascribed to internal bias fields caused by the oxygen vacancies. Supposing the oxygen vacancies should be randomly distributed, why is the Smax under positive field always larger than that under negative filed?

Re: The reviewer is correct that oxygen vacancies are expected to be randomly distributed in the absence of an applied electric field. However, their influence on the strain response is governed by the direction of the electric field applied during the first half-cycle of electrical loading. During this initial poling process, positively charged oxygen vacancies become associated with charged ferroelectric domain walls, forming relatively stable defect–domain-wall complexes. Once these defect–domain-wall associations are established, reorganizing them requires either a sufficiently large reverse electric field or elevated temperature, as reported previously [*Science* **378**,1125-1130(2022)]. Under the electric-field conditions employed in the present study, simply reversing the polarity of the applied field is insufficient to fully reverse the domain configuration or redistribute the oxygen vacancies. Consequently, the initial defect configuration is largely retained during subsequent electrical cycling.

Therefore, the maximum strain Smax may appear on either the positive or the negative field side, depending on the direction of the electric field applied during the first half-cycle of loading [*Journal of Materiomics* **11**, 101052 (2025)]. This behaviour reflects the history-dependent interaction between charged domain walls and oxygen vacancies, rather than a redistribution of oxygen vacancies during each measurement cycle. We have therefore revised the manuscript on page 9, accordingly.

4. Supplementary Fig.3 shows the grain size varies significantly from sample to sample. The grain size of the samples should be measured and the effect of grain on d33 should not be neglected.

Re: We thank the reviewer for this helpful comment. We have measured the average grain sizes from the SEM images and added the values to images. The grain size shows some variation among the samples, but it does not show any correlation with d_{33} (See the figure below), which suggests the effect of grain size on piezoelectric constant is not the dominant factor in this work. The d_{33} trend mainly follows the Ti-induced stabilization of polar order (Supplementary Fig. 8b) and d_{33} remains very low

for x ≤ 0.16 and increases markedly for x ≥ 0.18, consistent with the stronger remanent polarization, sharper switching features and enhanced local polar coherence. We have modified the manuscript on page 9, accordingly.

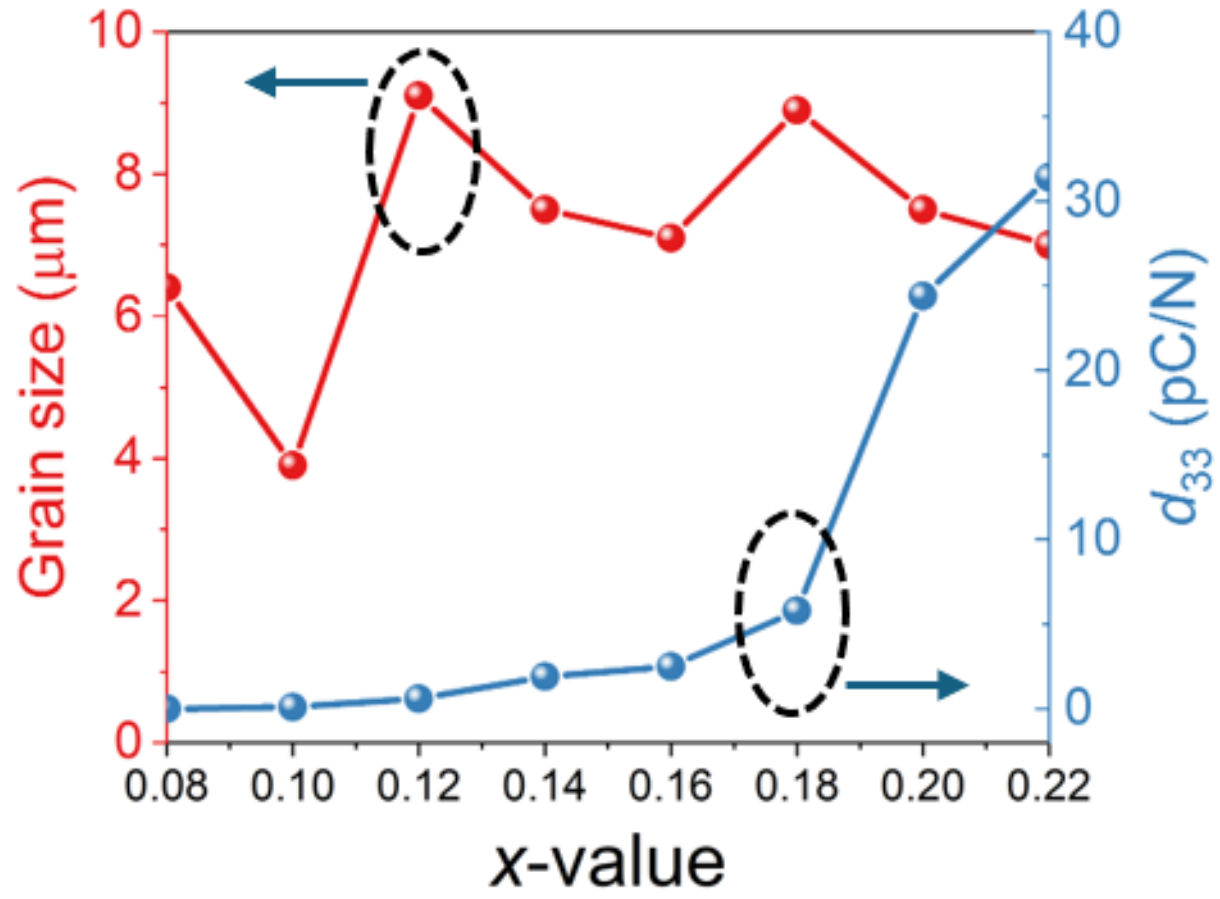

Figure 1. Composition dependent grain size and piezoelectric coefficient of the studied compositions.

Responses to Reviewers' Comments

We thank the reviewers for their helpful and constructive comments. Our responses to their specific points are provided below and all changes to the text are highlighted in the manuscript.

Reviewer #2:

I thank the authors for the substantial revision. The manuscript has improved, and the authors have addressed many of the earlier concerns carefully. The composition-resolved dataset remains interesting. I would appreciate clarification of a few points important for reproducibility and proportionate interpretation.

The distinction between total, reversible, and irreversible tunability is central. The Methods section could define each quantity and state the AC amplitude\frequency, DC loading\unloading sequence, dwell time, field-removal procedure, and zero-bias recovery interval. Please clarify how the 78.2 +- 1.0% total and 76.4% reversible values at x = 0.16 relate and how the 64.0% reversible value at x = 0.22 was obtained. Uncertainties would be useful if replicate reversible data exist; otherwise, these values could be identified as representative values.

Re: We thank the reviewer for this helpful comment. We now define total, reversible and irreversible tunability using the dielectric permittivity measured at the initial zero field, under DC bias and after field removal. The AC test condition, DC loading/unloading sequence, dwell time, field removal procedure and zero-bias recovery interval have also been added to the Methods. The value of 78.2 ± 1.0% refers to the total tunability measured from six independently prepared x = 0.16 specimens, whereas the 76.4% reversible tunability for x = 0.16 and 64.0% for x = 0.22 are representative values obtained from the reversible/irreversible separation shown in Fig. 3g. These representative reversible values are therefore not obtained by subtracting from the six-specimen mean. We have clarified this in the revised manuscript.

The DFT analysis needs clarification of the chemical configurations. The calculations use selected 3x3x3 periodic cells, but the Mg\Nb\Ti ordering model and the generation of the seven x = 0.16 configurations are unspecified; x = 0.08 and x = 0.22 remain represented by single configurations. Recent PMN–PT studies and the authors' observation that the broad spread among the seven x = 0.16 configurations itself demonstrates that the calculated polarization direction is sensitive to chemical configuration show that chemical disorder and residual short-range order influence local polar correlations. The cells also contain 3.70% Sm and no A-site vacancy rather than the nominal 2.5% Sm/A-site-vacancy chemistry. Thus, the calculations can

illustrate possible polarization directions for selected local configurations but cannot establish statistical isotropy, dynamics, or a unique composition-driven sequence. Providing the initial and relaxed structures, briefly describing configuration construction, and retaining a qualitative local-approximant interpretation would strengthen the paper. Terms such as "comparatively isotropic", "dynamically reconfigurable" and DFT-guided identification or design may need qualification. Additional sampling is needed only if the stronger mechanistic claims are retained.

Re: We thank the reviewer for this helpful comment. We have revised the DFT Methods and Supplementary Information to clarify how the selected chemical configurations were constructed, including the Mg/Nb/Ti arrangement and the seven additional configurations. We also clarified that the calculations use simplified local-approximant cells and do not represent full configurational averaging, A-site-vacancy chemistry, statistical isotropy or dipolar dynamics. The seven additional configurations are now discussed as demonstrating the sensitivity of the calculated polarization direction to local chemical configuration. Accordingly, terms such as “comparatively isotropic,” “dynamically reconfigurable” and “DFT-guided” have been modified throughout the manuscript. The DFT results are now retained only as qualitative illustrations of possible local polarization tendencies in selected configurations. The initial and relaxed structures have also been provided in the Source Data file.

The Amm2 assignments for PMNT16, PMNT22, and PMNT16v are not documented in the Methods or the Supplementary refinement table. I would appreciate provision of the available model-comparison and refinement information or, alternatively, a more cautious structural description. The newly added current-density data lack details regarding stabilization time and leakage compensation. Please state the measurement protocol and clarify whether conduction affects the P–E loops and remanent-polarization values. "Minimal hysteresis" should likewise be quantified or softened.

Re: We thank the reviewer for this helpful comment. We have revised the Methods section and Supplementary Information to document the structural refinement of the PMNT control compositions more clearly. Because PMNT16, PMNT22 and PMNT16v all show dielectric maxima above room temperature and ferroelectric-like *P-E*/*I-E* responses, their room-temperature XRD profiles were refined using the *Amm*2 ferroelectric average-structure model. To avoid overstatement, we now describe *Amm*2 as the refinement model used for these control compositions rather than as an unambiguous symmetry assignment. The corresponding refinement parameters for PMNT08, PMNT16, PMNT22 and PMNT16v are provided in Supplementary Table 1.

We have also revised the electrical measurement Methods to include the current-density measurement protocol. The current density was recorded at room temperature under a DC field of 2 kV mm^{-1} after a 50 s stabilization hold. We clarify that this stabilized DC current density is not directly equivalent to the current integrated during

the 1 Hz *P-E* loop measurement. The weak frequency dependence of the x = 0.16 *P-E* and *S-E* loops from 0.5 to 10 Hz, together with the discrete switching-current features in the *I-E* loops, suggests that steady conduction is not the dominant contribution to the measured *P-E* loops or remanent-polarization trends. Therefore, no explicit leakage subtraction was applied. To avoid overstatement, the term “minimal hysteresis” has been replaced with ‘low remanent polarization and small irreversible tunability’.

Please also correct the swapped Fig. 2h and Fig. 2i citations, the unit in Fig. 3b from μC m−2 to μC cm−2, and "composition tunning" in the abstract. Unless saturation is demonstrated for each composition, Pmax may be more appropriate than Ps.

Re: We thank the reviewer for pointing out these issues. We have corrected the swapped Fig. 2h and Fig. 2i citations, changed the unit in Fig. 3b to μC cm^{-2}, and corrected “composition tunning” to “composition tuning” in the abstract. We have now replaced P_s with P_{max}. The corresponding figure labels, captions and discussion have been revised accordingly.

Addressing these points would improve reproducibility and align the DFT and mechanistic interpretation with the evidence. I therefore recommend major revision.

Re: We thank the reviewer for the constructive assessment. We have revised the Methods and Results and Discussion sections to improve reproducibility, clarified the construction and limitations of the DFT configurations, and further moderated the mechanistic interpretation throughout the manuscript. These revisions align the interpretation more closely with the experimental and computational evidence.

Responses to Reviewers' Comments

We thank the reviewers for their helpful and constructive comments. Our responses to their specific points are provided below and all changes to the text are highlighted in the manuscript.

Reviewer #2:

I thank the authors for the careful revision. My previous substantive concerns have been addressed, and the interpretation is now appropriately aligned with the evidence. Only a small number of editorial corrections remain:

Re: We thank the reviewer for the positive assessment and for the careful editorial corrections. We have revised the manuscript and Reporting Summary accordingly.

Please standardize the instrument model numbers: "Agilent 4294" and "Agilent 4284" should be given by their full designations, 4294A and 4284A, consistently in the Methods and Reporting Summary.

Re: We thank the reviewer for pointing this out. We have standardized the instrument model numbers as Agilent 4294A and Agilent 4284A throughout the Methods and Reporting Summary.

Please verify and correct the Raman instrument identification and excitation wavelength: "ViaTM confocal Raman microscope" appears to be an incomplete or malformed model name, and the stated 432 nm wavelength is likely a typographical error for 532 nm. Please make these details consistent in the Methods and Reporting Summary.

Re: We thank the reviewer for this careful correction. We have verified the experimental record and corrected the Raman description to Renishaw inVia^{TM} confocal Raman microscope equipped with a 532 nm excitation laser. This has been made consistent in both the Methods and Reporting Summary.

Please revise the malformed opening of the Fig. 3a caption, "Current (Normalized current, I/Imax)-electric field", to a clear form such as "Normalized-current (I/Imax)-electric-field and P-E loops".*

Re: We have revised the Fig. 3 caption to clarify the description. It now reads: "Normalized-current (I/I_{max})-electric-field and *P–E* loops as functions of electric field".

Reference 5 should give 2024, rather than 2023, as the year associated with Nat. Biotechnol. 42, 448-457. Reference 50 should include the complete author list: S. L. Dudarev, G. A. Botton, S. Y. Savrasov, C. J. Humphreys and A. P. Sutton.

Re: We thank the reviewer for identifying these reference errors. We have corrected the year of Reference 5 to 2024 and updated Reference 50 to include the complete author list.
